# Focus, Newness and Their Combination: Processing of Information Structure in Discourse

**DOI:** 10.1371/journal.pone.0042533

**Published:** 2012-08-17

**Authors:** Lijing Chen, Xingshan Li, Yufang Yang

**Affiliations:** 1 Key Laboratory of Behavioral Science, Institute of Psychology, Chinese Academy of Sciences, Beijing, China; 2 Graduate University of Chinese Academy of Sciences, Beijing, China; University of Leicester, United Kingdom

## Abstract

The relationship between focus and new information has been unclear despite being the subject of several information structure studies. Here, we report an eye-tracking experiment that explored the relationship between them in on-line discourse processing in Chinese reading. Focus was marked by the Chinese focus-particle “shi", which is equivalent to the cleft structure “it was… who…" in English. New information was defined as the target word that was not present in previous contexts. Our results show that, in the target region, focused information was processed more quickly than non-focused information, while new information was processed more slowly than given information. These results reveal differences in processing patterns between focus and newness, and suggest that they are different concepts that relate to different aspects of cognitive processing. In addition, the effect of new/given information occurred in the post-target region for the focus condition, but not for the non-focus condition, suggesting a complex relationship between focus and newness in the discourse integration stage.

## Introduction

Information structure describes the manner in which information is packaged in a sentence, and is constrained by the context and the intention of information transfer. It is often specified by various dichotomies, such as background vs. focus, given vs. new information, and theme vs. rheme. Although these dichotomies have different connotations, they often overlap in meaning. For example, between background and focus, focus is often related to new information while background is not [1], [2]. In linguistic theories, the relation of focus with new information is still debated. The present study explores this issue in on-line discourse processing by observing the eye movements of readers.

In the dichotomy of background vs. focus, focus is the most prominent or emphasized constituent in a sentence [1], and the remaining part of the sentence is background. In the sentence (1b), “Tom" is emphasized by the cleft-structure and becomes the most prominent constituent, while “her" fades in the background. Focus is the information the writer/speaker wants to emphasize on and is often marked by certain linguistic devices. Besides syntactic structures like the cleft in example (1b), focus can be marked in other ways, such as the context of a wh-question (e.g., “Who helped Jane?"), focus-particles (e.g., “only", “always", and “shi" in Chinese), and accentuation in spoken language.

(1a) Context: Jane could not get the soap on the top of the shelf.

(1b) It was Tom who helped her.

With respect to the connotations of focus, Halliday [1] suggested that “what is focal is ‘new’ information" (p. 204) and further explained that “new" is what is factually new or contrastive. For example, in the sentence (1b), the focus “Tom" can be viewed as the answer to the implicit question “who helped her?", and thus provides new information. The focus that conveys new information is often called *information focus*. On the other hand, the sentence (1b) implies a contrastive meaning that “Tom helped her and nobody else helped her", and thus “Tom" is contrastive. The focus that implies contrast is called *contrastive focus* [3], [4]. Both information focus and contrastive focus are focus. However, new information also belongs to the dichotomy of given vs. new information.

In the dichotomy of given vs. new information, given information is what has been known with respect to the reader/listener's knowledge or the discourse model, while new information is the unknown information, and cannot be inferred from the context or shared by the writer/speaker and reader/listener. In a discourse, given information refers to the entity that has already occurred in a prior context, while new information is an entity that has not occurred previously [5]. For example, in the sentence (1b), “Tom" is new information, while “her", referring to “Jane" that occurred in the previous context, is given information. The given/new information in the discourse is also called “discourse-given/discourse-new". In the following text, the phrases “given-new information", “discourse-given" and “discourse-new" will be referred to as “newness", “given" and “new", respectively, for brevity.

The relationship between focus and new information is not straightforward, however. For example, in sentence (1b), if “Tom" is made given information by setting a context (e.g., “Jane went shopping with Tom and others. She could not get the soap on the top of the shelf."), “Tom" still remains the focus because it is emphasized by the cleft-structure. Thus, both new and given information can be the focus. This appears confusing, because if “new information" is an important part of focus, then “given information" should be excluded from it. To resolve this apparent contradiction, some linguists proposed theories to interpret focus and new information in a unitary framework. For example, according to Halliday [1], focus is “new" information, which is defined as textually non-derivable information (p. 205).The textually non-derivable information not only includes factually new information, but also includes “a matter of contrast with what has been said before or what might be expected" (p.206). Thus, if it is contrastive, the given information can be seen as functionally “new" information, and can be focused.

On the other hand, some linguists have recently tried to resolve this problem in a different way. They compared the difference between focus and newness, and suggested that they should be distinguished as two different concepts [6], [7]. For example, Féry and Krifka [6] redefined the concepts of focus and givenness, and argued that they should be categorized as different sub-concepts of information structure. Moreover, Selkirk [7] also suggested distinguishing contrastive focus from discourse-new information. Her suggestion has been supported by new evidence from acoustic studies showing the differences between contrastive focus and discourse-new information [8]–[11].

Although the relationship between focus and newness has been discussed plentifully in linguistics, psycholinguistic studies to date have focused mostly on the processing of information structure, while ignoring the relationship between focus and newness. A thorough study exploring the relationship and differences between them is lacking.

Several psycholinguistic studies have shown that focus have many advantages over non-focus. First, focus status affects referential processing. An entity in a focused position is more salient and more possible to be referred to than that in a non-focused position. Additionally, the pronoun or noun-phrase which refers to a focused antecedent is easier to resolve than the one that refers to a non-focused antecedent [12]–[15]. Second, focus status affects the depth of processing of concepts. Focused information is processed deeper and remembered better than non-focused information [16]–[18]. Additionally, the phoneme of focused information is detected quicker than that of non-focused information, and the change of focused information is more likely to be detected than that of non-focused information [16], [17], [19], [20]. Third, contrastive focus can guide the processing of syntax ambiguities. For example, using the focus-particle “only" to indicate potential contrast in a sentence can eliminate the syntax ambiguities of the “garden path" sentence [21]–[24]. Finally, focus may trigger integration effects. Electrophysiological studies have shown that focused element elicits a larger positivity, which reflects integration, than non-focused element [25]–[27]. It suggests that focused element may be integrated into the context immediately.

However, eye-tracking studies have provided inconsistent reports on on-line processing patterns of focus. Ward and Sturt [28] explored the on-line processing pattern of focused information using passages like “I couldn't decide *which seat* to take at the theatre. I hoped *the seat by the exit* would give me a good view. It turned out to be a wonderful evening's entertainment." The target word here is “the exit", which is set as the focus by the context “which seat" in the focus condition. Participants were asked to read two successive displays of such text and report whether there was a word changed in the second display (the experimenters changed one word in the second display for some trials) while their eye movements were monitored. Eye movements in the first display of the text reflect on-line processing. Surprisingly, no evident difference was found between on-line processing of focused and non-focused words. Morris and Folk [29] explored the on-line processing of focused information manipulated by it-clefts. They used sentences like “While the *waiter* watched, it was the *accountant* who balanced the ledger a second time." and “It was the *waiter* who watched while the *accountant* balanced the ledger a second time." The target words were “waiter" and “accountant", which were focused by the cleft-structure in the focus condition. Participants were asked to read such sentences for comprehension. They found that on-line processing of focused and non-focused words in the examples they used was different: their subjects fixated on focused words for shorter duration than on non-focused words. This effect was found in total reading time, which reflected the integration of information [30]. In addition, Birch and Rayner [31] also studied on-line processing of focused elements manipulated by it-clefts. They used passages like: “The tenants at the complex were sick and tired of all the noise coming from #204. It was the *landlady* who confronted the woman who lived there (*focus condition*). [The *landlady* confronted the woman who lived there (*non-focus condition*)]. She evicted the woman finally, to everyone's relief." The target word was “landlady", which was focused by the cleft-structure in the focus condition. They found similar results as Morris and Folk [29]– focused elements were fixated on for shorter duration than non-focused elements. They also found this effect in other eye movement measures, including first fixation duration, gaze duration, total time and total number of fixations, which suggested that lexical access and integration processes of focused elements were both facilitated compared to those of non-focused elements. One explanation for the apparent discrepancy in some of the results of these studies is that they differ in focus-marking devices (wh-question contexts or it-clefts), reading tasks (reading for detecting word change between two presentations of the text or for comprehension), and more importantly, definition of the information structure (confounding focus status and newness status or not). Further studies are needed to verify whether the discrepancies are indeed due to differences in study design.

Previous studies on given/new information have indicated that new information is processed slower than given information in comprehension and other behavioral tasks [32], [33]. This suggests that processing of new information is more difficult than of given information. Consistent with this, electrophysiological studies have shown that new information elicits a larger N400 than given information [34]–[38], suggesting that integration of new information must be more difficult than of given information. More importantly, eye-tracking studies have consistently shown that given information is processed quicker than new information during on-line discourse processing, which is called the repetition effect [30]. For instance, Raney and Rayner [39] monitored the eye movements of participants while they read texts twice. They found that during the second reading, the reading time decreased. Traxler, Foss, Seely, and Morris [40] had participants read sentences with repeated nouns, and found that the reading time for repeated nouns was less than that for new ones. Liversedge, Pickering, Clayes, and Branigan [41] found a similar repetition effect for verbs. Furthermore, Ledoux et al. [38] compared eye movements of participants as they read new and repeated proper names embedded in texts. They found that new names were fixated on longer than repeated ones. Thus, given information is processed faster during text reading irrespective of whether the target words are nouns, verbs or proper names.

In summary, eye tracking studies have shown that new information is fixated on longer than given information, while the results for focused information are inconsistent. Further studies are needed to clarify the effects of information structure on text reading for the following reasons. First, no study has directly compared the effects of focus and newness status in text reading. Moreover, no study has manipulated focus status and newness status simultaneously in a single experiment to examine their interactions. Thus whether there is an interaction between focus and newness is still unknown. Second, the effects of focus have been found to be different in different studies and further studies are needed to address the discrepancies. Finally, most studies have explored the effects of focus status and of newness status on reading in English. It is necessary to explore whether the effects are similar in other languages, such as Chinese.

In the current study, we compared the effects of focus status and newness status in Chinese reading in a single experiment using eye tracking technology. Focus status is marked by a focus-particle “shi" before the target word (Table 1). The focus-particle “shi" in Chinese is equivalent to the it-cleft structure in English [42]. The difference between them is that “shi" is only placed before the target word, but “it was… who…" is placed both before and after the target word. This feature of focus-particle “shi" allows us to compare the processing of post-target region between focus and non-focus conditions. Measuring the processing of post-target region is important as the effect in the target region can sometimes “spill over" onto the post-target region [30]. Additionally, the effect can sometimes occur in the post-target region instead of the target region, which suggests that the effect is related to a later stage of discourse processing such as integration [38], [41], [43]–[47]. Newness status is defined by whether the target word was present in the previous context or not. The present experiment uses proper name as the target word instead of identity noun (e.g., “landlord", “waiter" in previous studies, [29], [31]) to avoid activation of the knowledge of those words in the participant's memory. If focus and newness play different roles in reading, they should affect eye movements in different ways. Furthermore, if these two kinds of information structures affect different aspects of reading processes, their effects should be additive [48]. On the other hand, if they play similar roles in reading, they should affect eye movements in the same way and their effects should not be additive. Finally, because the eye movements in the target region often reflect the processing of the target words, while the eye movements in the post-target region are often related to the discourse integration, if focus and newness play different roles at different processing stages, they should affect eye movements differently between the target and post-target region.

**Table 1:** An Example of Experimental Materials*.

Condition	Context	Target sentence
**New/focus**	何仁正在劝说朋友们和他去郊游,根本不管天气预报说要变天。Heren was persuading his friends to go on an outing. (He) ignored that the weather forecast had predicted a bad weather.	这时候 *是* **钟莹** 理智地 反对 他。At that time *shi* **Zhongying** reasonably opposed him.At that time *it was* **Zhongying** *(who)* opposed him reasonably.
**New/non-focus**	何仁正在劝说朋友们和他去郊游,根本不管天气预报说要变天。Heren was persuading his friends to go on an outing. (He) ignored that the weather forecast had predicted a bad weather.	这时候 **钟莹** 理智地 反对 他。At that time **Zhongying** reasonably opposed him.At that time **Zhongying** opposed him reasonably.
**Given/focus**	何仁正在劝说钟莹他们去郊游,根本不管天气预报说要变天。Heren was persuading Zhongying and others to go on an outing. (He) ignored that the weather forecast had predicted a bad weather.	这时候 *是* **钟莹** 理智地 反对 他。At that time *shi* **Zhongying** reasonably opposed him.At that time *it was* **Zhongying** *(who)* opposed him reasonably.
**Given/non-focus**	何仁正在劝说钟莹他们去郊游,根本不管天气预报说要变天。Heren was persuading Zhongying and others to go on an outing. (He) ignored that the weather forecast had predicted a bad weather.	这时候 **钟莹** 理智地 反对 他。At that time **Zhongying** reasonably opposed him.At that time **Zhongying** opposed him reasonably.

* The bold words are the target words. The italicized words are the focus-particles. Brackets indicate that the words do not exist in the original Chinese materials. The bold, spaces, and italics are shown for illustration purposes only. They were not shown in the actual experiments.

## Materials and Methods

### Participants

36 undergraduate or graduate students (mean age: 23 years, range: 19–27; 17 males) participated in the experiment and were paid for their participation. All had normal or corrected-to-normal vision. This study was approved by the Institutional Review Board of the Institute of Psychology at the Chinese Academy of Science. All participants provided written informed consent.

### Materials

48 sets of three-sentence passages (Text S1) were constructed, and each passage had four versions. Each passage described a scene in everyday life, with two main characters referred to as Character A and B. Their names were composed of two Chinese characters. The genders of Character A and B were different. Character B was the target in the third sentence. For new information condition, he/she was first mentioned in the third sentence, and for old information condition, he/she had been introduced in the first sentence. Character A was the subject of the first sentence and was referred to by using two kinds of anaphoric expressions in the second sentence: overt pronoun (“she" or “he") and zero pronoun. A zero pronoun is an anaphoric expression in Chinese that has null grammatical subject for sentence. The default subject is the subject of the previous sentence. Yang et al. [49] showed that the processing of Chinese sentences employing overt pronouns and zero pronouns are the same. Hence, both anaphoric expressions were used to construct the second sentence. However, in each set of materials, only one kind of expression was used and the second sentences in all four versions were the same. In the third sentence, a focus-particle “shi" was placed before the target word for focus condition, but not for the non-focus condition.

48 sets of filler materials were used to prevent participants from guessing the pattern of experimental materials and reacting in a special way. The filler materials also described everyday lives and consisted of three-sentence passages with at least one character. For 12 of them, the focus-particles appeared in the first sentence; for another 12, the focus-particles appeared in the second sentence; for the remaining 24, there were no focus-particles in the passages. Besides, we constructed a set of 10 passages with the structure of the experimental passages and filler passages for use in practice trials. Furthermore, 37 comprehension questions that were relevant to the topics of the passages but not the target names were constructed.

These passages were divided into four lists and each passage was presented only once in each list. Each list contained 12 experimental passages per condition, all the 48 filler passages, and 10 practice passages. Participants were randomly assigned to one of the lists, and the number of participants in each list was equal.

### Apparatus

Eye movements of the participants were monitored using the Eyelink 2 eye tracker (SR Company). Sampling rate of the eye tracker was 250 Hz. The computer used for text presentation was TCL MF969A. Refresh frequency of the monitor was 85 Hz. Texts were presented in size 24 Chinese Songti font. The color of the characters was white and the background color was black. The visual angle of each character was 1.2°. Participants were seated 80 cm away from the display monitor, and their chins were placed on a bracket to eliminate head movements.

### Procedure

Participants were instructed to read short passages at their normal pace. Calibration was carried out before the experiment commenced, and recalibration was done during the experiment if necessary. Drift correction was conducted at the beginning of each trial. Each trial started with a small square appearing on the top left of the screen. Participants were required to fixate on the square for 100 ms before the square disappeared and a passage appeared with the first character at the location of the square. The entire passage was shown on the same display, with each sentence appearing on a separate line. Participants completed 10 practice trials to become familiar with the experiment before participating in the actual experiment. The paragraphs were displayed randomly. Participants read the passages at their own pace. When finished with a passage, they pressed a button and the text disappeared. Some trials were followed by a comprehension question to make sure that participants tried their best to understand the passages. Half of the questions required a YES response and half required a NO response. Participants used two handheld buttons to answer the questions. The viewing was binocular, but the movement of only one eye was monitored. Movement of the right eye was monitored for 34 of the 36 participants, while movement of the left eye was monitored for the other 2 participants due to bad calibration in their right eye. Each experiment lasted about 30 minutes. Participants were allowed to take breaks as needed.

### Data Analysis

Data obtained from four participants (3 females and 1 male) were not analyzed because the accuracy of their responses to the comprehension questions was lower than 80%. Average accuracy of the responses of the other subjects was 93.3%. Fixations less than 80 ms were merged to the nearest fixation point if the distance between them was less than 1 character. Other fixations shorter than 80 ms or longer than 800 ms were removed from the analyses, which excluded 0.78% of the fixations in total. 1.27% of the total trials were deleted due to occurrence of too many blinks or disruption of fixations. Four regions-of-interest (ROIs) were defined for each passage, as shown in the following example:

[1何仁正在劝说朋友们和他去郊游,根本不管天气预报说要变天_°_ 这时候(是)][2钟莹][3理智][4地反对他_°_ ]

[1 Heren was persuading his friends to go on an outing. (He) ignored that the weather forecast had predicted a bad weather. At that time (it was)][2 Zhongying] (who) [4 opposed him][3 reasonably].

The first region contained the first two sentences and the part before the target word in the third sentence, including the focus-particle in focus condition. The second region consisted of the target word. The third region consisted of the first two characters following the target word. In this example, the adverb “reasonably" came just after the target word in Chinese, but moved to the end of the sentence in English following proper language rules. The fourth region involved the remainder of the third sentence. It is noteworthy that although the focus-particle “shi" is translated into “it was…who…" in English, it was placed exactly before the target word and thus the characters in region 3 were the same in all four conditions.

The data of region 2 and region 3, which were the target and the post-target regions, respectively, were reported. Trials with no fixation on these two regions were excluded from our analysis. Measures of eye movement included first fixation duration, gaze duration, total time, and total number of fixations. First fixation duration is the duration of the first fixation in the region. Gaze duration is the time spent in the region from fixation coming into the region to moving off it. Total time is the sum of all fixation durations in the region. Total number of fixations indicates the number of all fixations in the region [30], [31], [43]. First fixation duration and gaze duration are usually thought to reflect early lexical access and encoding, while total time and total number of fixations are usually thought to reflect the later integration stage in reading [31], [43]. For each data set, two ANOVAs by participants and by items were conducted, treating participants (*F*_1_) and items (*F*_2_), respectively, as random factors.

## Results

Table 2 and Table 3 show the descriptive statistics for the target region and the post-target region, respectively.

**Table 2:** Eye Movement Measures for the Target Region.

	New Information	Given Information
	M(SE)	M(SE)
*First fixation duration (ms)*		
Focus	256(8.4)	240(7.3)
Non-focus	273(8.7)	254(8.6)
*Gaze duration (ms)*		
Focus	288(11.8)	272(12.6)
Non-focus	337(16.4)	292(13.6)
*Total time (ms)*		
Focus	504(32.4)	418(28.2)
Non-focus	562(38.5)	456(24.3)
*Total number of fixations*		
Focus	1.86(.14)	1.59(.13)
Non-focus	2.01(.16)	1.75(.11)

**Table 3:** Eye Movement Measures for the Post-target Region.

	New Information	Given Information
	M(SE)	M(SE)
*First fixation duration (ms)*		
Focus	259(7.8)	244(6.6)
Non-focus	245(8.9)	241(7.4)
*Gaze duration (ms)*		
Focus	307(13.2)	263(8.2)
Non-focus	279(14.4)	260(8.6)
*Total time (ms)*		
Focus	451(22.6)	377(18.2)
Non-focus	411(19.0)	376(18.2)
*Total number of fixations*		
Focus	1.55(.09)	1.38(.10)
Non-focus	1.48(.08)	1.38(.08)

### Target Region

For first fixation duration, focused information took less time to read than non-focused information [F1(1,31) = 4.72, MSe = 7518, p<.05; F2(1,47) = 9.64, MSe = 14060, p<.01], while new information took longer to read than given information [F1(1,31) = 9.78, MSe = 9574, p<.01; F2(1,47) = 13.61, MSe = 17499, p = .001]. There was no significant interaction between focus and newness (both Fs<1). These results indicated that the processing pattern of new information was quite different from that of focused information.

These effects were reliable for gaze duration, total time and total number of fixations. For gaze duration, focused information took less time to read than non-focused information [F1(1,31) = 7.5, MSe = 37881, p = .01; F2(1,47) = 17.52, MSe = 63839, p<.001], while new information took longer to read than given information [F1(1,31) = 8.64, MSe = 29646, p<.01; F2(1,47) = 13.56, MSe = 54506, p = .001], and no significant interaction was observed [F1(1,31) = 3.67, MSe = 7081, p = .065, F2<1]. For total time, focused information took less time to read than non-focused information [F1(1,31) = 7.66, MSe = 74498, p<.01; F2(1,47) = 9.45, MSe = 106173, p<.01], and new information took longer to read than given information [F1(1,31) = 24.85, MSe = 297413, p<.001; F2(1,47) = 45.45, MSe = 425351, p<.001], and no interaction was detected between them (Fs<1). For total number of fixations, focused information was fixated on less than non-focused information [F1(1,31) = 8.81, MSe = 0.781, p<.01; F2(1,47) = 8.94, MSe = 1.156, p<.01], while new information was fixated on more than given information [F1(1,31) = 16.63, MSe = 2.311, p<.001; F2(1,47) = 17.03, MSe = 3.548, p<.001], and no interaction was detected between them (Fs<1).

### Post-target Region

For first fixation duration, the main effect of focus was not significant [F1 = 2.57, MSe = 2261, p = .119; F2 = 2.04, MSe = 3128, p = .160], but the fixation duration for the focus condition tended to be longer than that for the non-focus condition, which was different from the focus effect in the target region. The words in the new information condition were fixated on longer than those in the given information condition [F1(1,31) = 3.00; MSe = 2945; p = .093; F2(1,47) = 5.22; MSe = 8099; p<.05]. However, these effects were qualified by the interaction between focus and newness (significant by items) [F1(1,31) = 1.47, MSe = 1058, p = .235; F2(1,47) = 4.15, MSe = 5515, p<.05]: for the focus condition, the fixation duration in the new information condition was significantly (by items) longer than that in the given information condition [F(1,31) = 3.92, MSe = 3767, p = .057; F2(1,47) = 7.42, MSe = 13490, p<.001], while this effect was not significant for the non-focus condition (both Fs<1). This indicated that the newness effect in the post-target region occurred only in the focus condition.

These effects were also valid for gaze duration and total time. For gaze duration, the fixation duration for the focus condition was significantly (by participant) longer than that for the non-focus condition [F1(1,31) = 7.35, MSe = 7336, p<.05; F2(1,47) = 3.65, MSe = 12513, p = .062], and the fixation duration for the new information condition was significantly longer than that for the given information condition [F1(1,31) = 13.96, MSe = 31595, p = .001; F2(1,47) = 21.23, MSe = 60776, p<.001]. Moreover, these effects were qualified by the interaction between focus and newness [F1(1,31) = 5.44, MSe = 4888, p<.05; F2(1,47) = 6.39, MSe = 18408, p<.05]: for the focus condition, the fixation duration in the new information condition was significantly longer than that in the given information condition [F(1,31) = 17.86, MSe = 30669, p<.001; F2(1,47) = 22.02, MSe = 73041, p<.001], while for the non-focus condition, the difference between the two conditions was not significant [F(1,31) = 4.03, MSe = 5814, p = .054; F2(1,47) = 2.53, MSe = 6144, p = .118].

For total time, the reading time for the focus condition was significantly (by participant) longer than that for the non-focus condition [F1(1,31) = 4.27, MSe = 13264, p<.05; F2(1,47) = 3.14, MSe = 28739, p = .083] and the reading time for the new information condition was significantly longer than that for the given information condition [F1(1,31) = 13.55, MSe = 93799, p = .001; F2(1,47) = 14.62, MSe = 133510, p<.001]. These effects were also qualified by the interaction between focus and newness (significant by items) [F1(1,31) = 2.59, MSe = 12423, p = .118; F2(1,47) = 4.64, MSe = 27004, p<.05]: for the focus condition, the fixation duration in the new information condition was significantly longer than that in the given information condition [F(1,31) = 12.38, MSe = 87246, p = .001; F2(1,47) = 15.29, MSe = 140301, p<.001], while for the non-focus condition, the difference between the two conditions was not significant [F(1,31) = 4.04, MSe = 18975, p = .053; F2(1,47) = 3.50, MSe = 20213, p = .068].

The total number of fixations for the new information condition was significantly higher than that for the given information condition [F1(1,31) = 6.99, MSe = 0.605, p<.05; F2(1,47) = 6.27, MSe = 0.867, p<.05]. However, neither the main effect of focus nor the interaction between focus and newness was significant (Fs<1). It appears that the interaction is present only in time measures but not in number measures.

## Discussion

The present study investigated the difference and relationship between focus and newness by recording eye movements of Chinese readers as they read short passages. Two important results were observed. First, our results on first fixation duration, gaze duration, total reading times and total number of fixations in the target region showed that the processing pattern of focus was different from that of newness. Focused information was processed more quickly than non-focused information, while new information was processed more slowly than given information. Second, our results on first fixation duration, gaze duration and total reading times in the post-target region revealed an interaction between newness and focus. For non-focused sentences, there was no significant difference between new and given information. For focused sentences, however, the region in the new information condition was fixated on longer than that in the given information condition. These results indicated that focus modulated the newness effect in the discourse integration stage.

We observed different effects on eye movement measures between focus and newness. Our results showed that new information was read more slowly than given information, whereas focused information was read more quickly than non-focused information. Here, the focus, which was indicated by the focus-particle “shi", is contrastive focus. Thus, based on our data, it can be claimed that contrastive focus and new information are processed differently during reading. This finding is inconsistent with the theories that suggest contrastive focus and new information are subjected to similar linguistic principles [1], [2], [50]. To be consistent with these theories, contrastive focus and new information should show similar processing patterns. However, our results showed the opposite. These theories are based on semantic analysis [1], [50] or phonological analysis [2]. Thus, though our results are inconsistent with their predictions, it is likely that they only predict the patterns of semantic interpretation and prosodic production, which need not to be similar to on-line processing patterns. Moreover, the definitions of “new information" are different in different theories, which reduce the comparability of them.

On the other hand, however, our results are consistent with the theories that claim that focus and newness are different concepts and suggest making a distinction between contrastive focus and new information. As an example, Féry and Krifka [6] proposed a three-way distinction between focus, givenness and topic, which defined focus and givenness as different categories. They suggested that focus and givenness are independent concepts of information structure, and there is no strict relation between focus and new information (for example, given information can also be focused on). Furthermore, Selkirk [7] proposed a three-way distinction in which contrastive focus, discourse-new and discourse-given are three parallel concepts that make up the whole information structure. This distinction is also supported by acoustic studies which showed the prosodic differences among contrastive focus, discourse-new, discourse-given and second occurrence focus (SOF, a repeated focused entity which has appeared in a previous context) [8]–[11]. While these acoustic studies revealed the precise acoustic differences between focus and newness from the speaker's perspective, we have identified the differences between focus and newness in on-line processing from the perspective of comprehension.

From the standpoint of comprehension, our results can be explained in terms of differences in the cognitive processes involved. That focused information was found to be processed more quickly than non-focused information may reflect the facilitation in processing and integration for focused information, which may be realized by allocating more attention to focused information. This is consistent with the findings of Birch and Rayner [31] and Morris and Folk [29]. Previous studies have indicated that, in sentence or discourse comprehension, focus-particle is computed rapidly to affect discourse processing [44]. Givón [51] proposed a “mental processing instructions" theory and suggested that linguistic cues function as mental processing instructions and inform the reader/listener of the importance of concepts and the amount of attention to allocate to them. It is likely that the focus-particle brings more attention to the focused element in a similar manner. Indeed, other studies have suggested that focused information gains more attention than non-focused information [31], [52], [53]. On the other hand, new information was processed more slowly than given information. This result may reflect the difficulty in memory retrieval and updating of new information. Previous studies have indicated that this difficulty is influenced both by lexical access [36], [38], [54]–[57] and discourse integration stages [34], [35], [37], [38], which are confirmed by our results of the early processing measures (first fixation duration, gaze duration) and later integration measures (total time, total number of fixations). In summary, the focus effect is likely a result of more attention being allocated to focused information, whereas the newness effect likely reflects the difficulty in memory retrieval and updating of new information. This difference in cognitive processing may be responsible for their differences in processing patterns.

The target word in the given information condition is also a repeated word. This manipulation may raise the question whether the newness effect observed in the present study is the given-new effect in information structure, or just a word repetition effect which facilitated word recognition. While the exact underlying mechanism of this effect is unclear, the most plausible hypothesis is that both of them occur. Word repetition may facilitate its recognition. However, this effect was not only based on the facilitation of word form recognition, but also on the facilitation of the concept activation in the discourse model. Several studies have reviewed the different theories about the mechanism of repetition effects and suggested that the word repetition effect in text can be explained by both “lexical-level" and “text-level" facilitation [54], [56], [57]. Moreover, the ERP results that new information elicited a larger N400 than repeated information, which reflects lexical integration, also suggested that the repetition effect was related to the discourse integration, not just to word recognition [35], [37], [38]. More importantly, the effect is not only observed on repeated words, but also on synonymy [37], further suggesting that the effect isn't just based on word form processing. In addition, a similar N400 effect was found for new-given information in spoken discourse processing [34], in which the participants listened to the passages instead of reading them and there was no visual word form repetition. These results also point to the same explanation.

However, our results are different from those of some previous studies. Ward and Sturt [28] showed that there was no difference between the on-line processing of focused and non-focused information, which is inconsistent with our results. This discrepancy may be explained as follows. First, just as we did, they found that focused information was processed more quickly than non-focused information in some measures. However, because some target words in their focus condition had appeared in the preceding context while all the target words in their non-focus condition were new, they proposed that the effect might have been a repetition effect and there was no inherent difference between focused and non-focused information. However, though the effect could be caused by the repetition, it could also be caused by the focus. The possibility of focus effect could not be excluded. Second, they asked participants to read the text and complete a text-change detection task. The text-change detection task, in which participants have to decide whether there is any word changed in two versions of the same passage, is similar to proofreading. Proofreading is different from reading-for-comprehension. This difference can influence eye movement measures. For example, first fixation and gaze duration have been reported to be longer during proofreading than during reading for comprehension [58]. Third, focus marking is not identical between the two studies. The wh-question context which was used to indicate focus in their study was somewhat different from the focus-particle “shi" (or it-cleft structure) used in our study. This is an important consideration, as it has been shown that not all kinds of focus elicit the same processing pattern [25]. For example, an ERP study showed that violating the exhaustiveness of focus in on-line processing elicited N400 (400–600 ms) for cleft-focus, but elicited positivity (600–800 ms) for *only*-focus [59]. Similarly, an eye movement study also showed that the effect of contrastive focus was delayed for *even*-focus compared to *only*-focus [60].

Gordon et al. [61] found that a prominent antecedent should not be referred to in the form of repeated name as it can cause processing difficulty. This is called repeated name penalty. Repeated name penalty reflects the coreference rule constraint that a prominent antecedent should be referred to in reduced form (e.g., pronoun, zero pronoun). This effect does not occur in a non-prominent antecedent [38], [61]. We did not observe the repeated name penalty in our study. The target words in the given information condition were repeated names in our experiments. However, their antecedents were non-prominent in the first sentence. This may explain why no repeated name penalty was observed.

No study has yet examined the effect of focus in the post-target region. This may have resulted from the restriction of focus-marking in English. However, the results in the post-target region are helpful to understand the processing of focus. The effects occurring in the post-target region have been observed in many eye movement studies [38], [41], [43]–[47], especially the ones that examined anaphor processing [38], [45] and context modulation effects [43], [46], [47], which suggests an association of the post-target region processing with anaphor and discourse integration. In the present study, the results obtained for the post-target region were very different from that for the target region. An interaction between focus and newness was observed in the post-target region. For focus condition, the post-target region in the new information condition was processed slower than that in the given information condition. For non-focus condition, however, no significant effects were observed. It appears that although focus status and newness status affect on-line processing of target words differently and independently, they affect the anaphor and discourse integration in an interactive way. This effect may be caused by the conflict between the integration effect triggered by focus and the difficulty of integrating new information into the discourse context. Discourse processing is an incremental process [62] in which information is integrated into the context on-line. Given information, which has an antecedent in a previous context, is integrated into the context easily. However, new information, if without suitable given information as “bridge", will be difficult to integrate into the context. Discourse integration, on the other hand, is flexible; the integration of new information can be delayed until enough information has been received. This is why the newness effect did not occur in the non-focus condition. On the other hand, since focus triggers integration effects [25], [26], focused information is inclined to be integrated into the context immediately. However, if new information also has to be integrated into the context immediately, the difficulty of integration is greatly amplified. This may be the reason why the newness effect occurred in focused sentences.

On the other hand, the effect occurs in the post-target region as well reflects the processing of the post-target word itself. Thus, there is another explanation for the interaction. In the present study, post-focused information took longer to read than post-non-focused information for new information, while this effect disappeared for given information. This suggests that the post-focused constituent is more difficult to process than the post-non-focused constituent, but this effect only appears in new information. This result can be explained by the “enhancement and suppression" mechanism [16]. Sanford et al. [16] proposed that the cleft structure results in enhancement and suppression effects—it not only enhances the processing of the cleft element (i.e., focused information), but also suppresses the processing of other constituents of the sentence to pretest the processing of focused information. The processing difficulty of the post-focused constituent observed in our study may reflect suppression from the cleft-structure, which tries to pretest the processing of focused information. However, this effect disappears in the given information condition, which may be because the processing of given information is easier than of new information. The processing of given-focused information may be adequate without suppressing the processing of the post-focused constituent. This dissociation of suppression in different conditions may explain a question left unanswered in Sanford et al. [16]. In their study, their first experiment provided evidence to support the enhancement and suppression model, but their second experiment only supported the enhancement part of this model. Based on our results, it appears that the execution of suppression depends on the situation—if the processing of the target word is simple enough, suppression is not executed.

Most studies on the effects of focus status and newness status have been conducted in English reading. The results of the current study show that the effects of these information structures are robust across languages. The similarities in the results obtained in Chinese reading and English reading suggest that high-level processing of languages is done similarly irrespective of the language. Since words are separated by spaces in English but not in Chinese, the eye-movement patterns in Chinese text reading are slightly different from that in English text reading [63]. These differences are likely caused by low-level visual features in Chinese text. However, aspects of high-level language processing such as information structure processing are likely similar in English and Chinese. This further suggests that information structure is a general language device shared by English and Chinese and likely also by various other languages and cultures.

In the present study, fictional proper names were used as the target words to avoid the activation of world knowledge of those words. Accordingly, the results were interpreted in the framework of linguistics and only consider the influence of information structure. However, considering that in the daily conversation, words generally activate world knowledge, the interaction between the activation/integration of world knowledge and the effect of information structure should be further explored. One possible way is to explore the effect on identity nouns which may activate relevant world knowledge. The other possible way is to explore the effect on real names, such as the names of pop stars, which may recall the personal information about them.

In summary, the eye movement patterns of two dichotomies of information structure in discourse reading were compared in this study. Our results show that they have different on-line processing patterns: focused information is processed faster than non-focused information, while new information is processed slower than given information. Moreover, the processing patterns in the post-target region are very different from that in the target region. Newness and focus show interaction in their effect on discourse processing, suggesting a complex relationship between them in the discourse integration stage.

## Supporting Information

Text S1 **The experimental materials and filler materials.**
(RTF)
