# Supplementary material for: Focus, Newness and Their Combination: Processing of Information Structure in Discourse
Source: PLoS One. 2012 Aug 17;7(8):e42533. doi: 10.1371/journal.pone.0042533 (PMC3422350; doi:10.1371/journal.pone.0042533)
Supplement: Text S1 — The experimental materials and filler materials. (RTF) [file pone.0042533.s001.rtf]

Experiment Materials
£¨for each item, the order of the four passages is: 1st, new-focus condition; 2nd, new-nonfocus condition; 3rd, given-focus condition; 4th, given-nonfocus condition; 5th, comprehension question--if any£©

Items 1
ÖÜÕä´ÇµôÄÇ·ÝÐ½Ë®Î¢±¡µÄ¹¤×÷£¬ÔÚÐ¡ÇøÃÅ¿Ú¿ªÁË¸öÔÓ»õµê¡£½Ö·»¶¼ËµÊÇÔ¬´Ï°µÖÐ°ïÖúÁËËý¡£
ÖÜÕä´ÇµôÄÇ·ÝÐ½Ë®Î¢±¡µÄ¹¤×÷£¬ÔÚÐ¡ÇøÃÅ¿Ú¿ªÁË¸öÔÓ»õµê¡£½Ö·»¶¼ËµÔ¬´Ï°µÖÐ°ïÖúÁËËý¡£
ÖÜÕäÄÇÌì°Ý·ÃÁËÔ¬´ÏÖ®ºó£¬ÔÚÐ¡ÇøÃÅ¿Ú¿ªÁË¸öÔÓ»õµê¡£½Ö·»¶¼ËµÊÇÔ¬´Ï°µÖÐ°ïÖúÁËËý¡£
ÖÜÕäÄÇÌì°Ý·ÃÁËÔ¬´ÏÖ®ºó£¬ÔÚÐ¡ÇøÃÅ¿Ú¿ªÁË¸öÔÓ»õµê¡£½Ö·»¶¼ËµÔ¬´Ï°µÖÐ°ïÖúÁËËý¡£
ÖÜÕä¿ªÁËÒ»¸ö·þ×°µê¡£

2
·¶Ó¯ÄÜ¹»È¡µÃ³É¹¦Ó¦¸Ã¸ÐÐ»ÃüÔË£¬µ±ÄêËýµÄÃæÊÔ±íÏÖ²¢²»ºÃ¡£ºóÀ´ÊÇÉò²¨Á¦ÅÅÖÚÒéÂ¼È¡ÁËËý¡£
·¶Ó¯ÄÜ¹»È¡µÃ³É¹¦Ó¦¸Ã¸ÐÐ»ÃüÔË£¬µ±ÄêËýµÄÃæÊÔ±íÏÖ²¢²»ºÃ¡£ºóÀ´Éò²¨Á¦ÅÅÖÚÒéÂ¼È¡ÁËËý¡£
·¶Ó¯ÄÜ¹»È¡µÃ³É¹¦Ó¦¸Ã¸ÐÐ»Éò²¨£¬µ±ÄêËýµÄÃæÊÔ±íÏÖ²¢²»ºÃ¡£ºóÀ´ÊÇÉò²¨Á¦ÅÅÖÚÒéÂ¼È¡ÁËËý¡£
·¶Ó¯ÄÜ¹»È¡µÃ³É¹¦Ó¦¸Ã¸ÐÐ»Éò²¨£¬µ±ÄêËýµÄÃæÊÔ±íÏÖ²¢²»ºÃ¡£ºóÀ´Éò²¨Á¦ÅÅÖÚÒéÂ¼È¡ÁËËý¡£
·¶Ó¯µ±ÄêµÄÃæÊÔ±íÏÖ·Ç³£ºÃ¡£

3
ºúÁé½ñÌìË­¶¼²»Ïë¼û£¬ËýÕâ»á¶ùÇéÐ÷·Ç³£µÍÂä¡£ÒòÎªÊÇÌï¾üÑÏÀ÷µØÅúÆÀÁËËý¡£
ºúÁé½ñÌìË­¶¼²»Ïë¼û£¬ËýÕâ»á¶ùÇéÐ÷·Ç³£µÍÂä¡£ÒòÎªÌï¾üÑÏÀ÷µØÅúÆÀÁËËý¡£
ºúÁé¿´¼ûÌï¾ü×ªÉí¾Í×ß£¬ËýÕâ»á¶ùÇéÐ÷·Ç³£µÍÂä¡£ÒòÎªÊÇÌï¾üÑÏÀ÷µØÅúÆÀÁËËý¡£
ºúÁé¿´¼ûÌï¾ü×ªÉí¾Í×ß£¬ËýÕâ»á¶ùÇéÐ÷·Ç³£µÍÂä¡£ÒòÎªÌï¾üÑÏÀ÷µØÅúÆÀÁËËý¡£

4
ÂÞµ¼ÑÝ×î½ü×¼±¸ÅÄÒ»²¿µçÓ°£¬¿ÉÊÇËûÔõÃ´Ò²ÕÒ²»µ½ºÏÊÊµÄÖ÷ÑÝ¡£ºóÀ´ÊÇÅËÁáÈÈÐÄµØ°ïÖúÁËËû¡£
ÂÞµ¼ÑÝ×î½ü×¼±¸ÅÄÒ»²¿µçÓ°£¬¿ÉÊÇËûÔõÃ´Ò²ÕÒ²»µ½ºÏÊÊµÄÖ÷ÑÝ¡£ºóÀ´ÅËÁáÈÈÐÄµØ°ïÖúÁËËû¡£
ÂÞµ¼ÑÝ¸úÅËÁáËµÒªÅÄÒ»²¿µçÓ°£¬¿ÉÊÇËûÔõÃ´Ò²ÕÒ²»µ½ºÏÊÊµÄÖ÷ÑÝ¡£ºóÀ´ÊÇÅËÁáÈÈÐÄµØ°ïÖúÁËËû¡£
ÂÞµ¼ÑÝ¸úÅËÁáËµÒªÅÄÒ»²¿µçÓ°£¬¿ÉÊÇËûÔõÃ´Ò²ÕÒ²»µ½ºÏÊÊµÄÖ÷ÑÝ¡£ºóÀ´ÅËÁáÈÈÐÄµØ°ïÖúÁËËû¡£

5
Ç°¼¸ÌìËïÇ«³ï±¸¿ªÑ§Ã¦µÃÉú²¡ÁË£¬¿ªÑ§ÄÇÌìËû¶¼Ã»·¨È¥Ó­½ÓÐÂÉúÁË¡£ÐÒºÃÊÇËÕæ¼Ï¸ÐÄµØÕÕ¹ËÁËËû¡£
Ç°¼¸ÌìËïÇ«³ï±¸¿ªÑ§Ã¦µÃÉú²¡ÁË£¬¿ªÑ§ÄÇÌìËû¶¼Ã»·¨È¥Ó­½ÓÐÂÉúÁË¡£ÐÒºÃËÕæ¼Ï¸ÐÄµØÕÕ¹ËÁËËû¡£
Êî¼ÙËÕæ¼ÂÖÐÝÊ±ËïÇ«Ã¦µÃÉú²¡ÁË£¬¿ªÑ§ÄÇÌìËû¶¼Ã»·¨È¥Ó­½ÓÐÂÉúÁË¡£ÐÒºÃÊÇËÕæ¼Ï¸ÐÄµØÕÕ¹ËÁËËû¡£
Êî¼ÙËÕæ¼ÂÖÐÝÊ±ËïÇ«Ã¦µÃÉú²¡ÁË£¬¿ªÑ§ÄÇÌìËû¶¼Ã»·¨È¥Ó­½ÓÐÂÉúÁË¡£ÐÒºÃËÕæ¼Ï¸ÐÄµØÕÕ¹ËÁËËû¡£
ËïÇ«Õâ¼¸ÌìÉú²¡ÁË¡£

6
Ò¶×Ï¶À×ÔÒ»ÈËÈ¥³¬ÊÐ¹ºÎï£¬ËýÏÖÔÚ±ÈÒÔÇ°¿ªÀÊ¶àÁË¡£¿´À´ÊÇ¶­ÌÎ³É¹¦µØ¸Ä±äÁËËý¡£
Ò¶×Ï¶À×ÔÒ»ÈËÈ¥³¬ÊÐ¹ºÎï£¬ËýÏÖÔÚ±ÈÒÔÇ°¿ªÀÊ¶àÁË¡£¿´À´¶­ÌÎ³É¹¦µØ¸Ä±äÁËËý¡£
Ò¶×Ï¸æ±ð¶­ÌÎÈ¥³¬ÊÐ¹ºÎï£¬ËýÏÖÔÚ±ÈÒÔÇ°¿ªÀÊ¶àÁË¡£¿´À´ÊÇ¶­ÌÎ³É¹¦µØ¸Ä±äÁËËý¡£
Ò¶×Ï¸æ±ð¶­ÌÎÈ¥³¬ÊÐ¹ºÎï£¬ËýÏÖÔÚ±ÈÒÔÇ°¿ªÀÊ¶àÁË¡£¿´À´¶­ÌÎ³É¹¦µØ¸Ä±äÁËËý¡£

7
¸ß½ã½ã´ø¶ÓÔ±È¥É­ÁÖ²É¼¯±ê±¾£¬Í»È»Ëý²»Ð¡ÐÄ²Èµ½Ò»Ìõ´óòþÉß¡£Î£¼±Ê±¿ÌÊÇÎºÃùÓÂ¸ÒµØ¾ÈÁËËý¡£
¸ß½ã½ã´ø¶ÓÔ±È¥É­ÁÖ²É¼¯±ê±¾£¬Í»È»Ëý²»Ð¡ÐÄ²Èµ½Ò»Ìõ´óòþÉß¡£Î£¼±Ê±¿ÌÎºÃùÓÂ¸ÒµØ¾ÈÁËËý¡£
¸ß½ã½ã´øÎºÃùËûÃÇÈ¥É­ÁÖ²É¼¯±ê±¾£¬Í»È»Ëý²»Ð¡ÐÄ²Èµ½Ò»Ìõ´óòþÉß¡£Î£¼±Ê±¿ÌÊÇÎºÃùÓÂ¸ÒµØ¾ÈÁËËý¡£
¸ß½ã½ã´øÎºÃùËûÃÇÈ¥É­ÁÖ²É¼¯±ê±¾£¬Í»È»Ëý²»Ð¡ÐÄ²Èµ½Ò»Ìõ´óòþÉß¡£Î£¼±Ê±¿ÌÎºÃùÓÂ¸ÒµØ¾ÈÁËËý¡£
´ó¼ÒÈ¥É­ÁÖ²É¼¯±ê±¾¡£

8
³ÂÏÈÉú¶ÔÊé·¨ÒÕÊõ·Ç³£³ÕÃÔ£¬ËûÒ»ÏòÈÏÎª×Ô¼ºµÄ¼øÉÍË®Æ½Ò»Á÷¡£²»ÁÏÊÇÌÆÑþºÝºÝµØ´ò»÷ÁËËû¡£
³ÂÏÈÉú¶ÔÊé·¨ÒÕÊõ·Ç³£³ÕÃÔ£¬ËûÒ»ÏòÈÏÎª×Ô¼ºµÄ¼øÉÍË®Æ½Ò»Á÷¡£²»ÁÏÌÆÑþºÝºÝµØ´ò»÷ÁËËû¡£
³ÂÏÈÉú´øÌÆÑþÈ¥²Î¹ÛËûµÄÊé·¨×÷Æ·£¬ËûÒ»ÏòÈÏÎª×Ô¼ºµÄ¼øÉÍË®Æ½Ò»Á÷¡£²»ÁÏÊÇÌÆÑþºÝºÝµØ´ò»÷ÁËËû¡£
³ÂÏÈÉú´øÌÆÑþÈ¥²Î¹ÛËûµÄÊé·¨×÷Æ·£¬ËûÒ»ÏòÈÏÎª×Ô¼ºµÄ¼øÉÍË®Æ½Ò»Á÷¡£²»ÁÏÌÆÑþºÝºÝµØ´ò»÷ÁËËû¡£
³ÂÏÈÉú³ÕÃÔÓÚ»æ»­ÒÕÊõ¡£

9
ÐìÑà¾­³£µ½¸ô±ÚÄÇ¼Ò²Í¹Ý³Ô·¹£¬½ñÌìËý³ÔÍê·¹ºó±»¸æÖª²»ÓÃ½áÕË¡£Ô­À´½ñÌìÊÇ²ÌÅôÃâ·ÑÕÐ´ýËý¡£
ÐìÑà¾­³£µ½¸ô±ÚÄÇ¼Ò²Í¹Ý³Ô·¹£¬½ñÌìËý³ÔÍê·¹ºó±»¸æÖª²»ÓÃ½áÕË¡£Ô­À´½ñÌì²ÌÅôÃâ·ÑÕÐ´ýËý¡£
ÐìÑà¾­³£µ½²ÌÅô¼ÒµÄ²Í¹Ý³Ô·¹£¬½ñÌìËý³ÔÍê·¹ºó±»¸æÖª²»ÓÃ½áÕË¡£Ô­À´½ñÌìÊÇ²ÌÅôÃâ·ÑÕÐ´ýËý¡£
ÐìÑà¾­³£µ½²ÌÅô¼ÒµÄ²Í¹Ý³Ô·¹£¬½ñÌìËý³ÔÍê·¹ºó±»¸æÖª²»ÓÃ½áÕË¡£Ô­À´½ñÌì²ÌÅôÃâ·ÑÕÐ´ýËý¡£

10
Ò»´óÔçÍõÃ÷¾Í±³ÆðÊé°üÉÏÑ§È¥ÁË£¬Ëû¸Õ´ÓÒ»³¡´ó²¡ÖÐ»Ö¸´¹ýÀ´¡£½ñÌìÊÇÓÚÙ»¸ºÔðÕÕ¹ËËû¡£
Ò»´óÔçÍõÃ÷¾Í±³ÆðÊé°üÉÏÑ§È¥ÁË£¬Ëû¸Õ´ÓÒ»³¡´ó²¡ÖÐ»Ö¸´¹ýÀ´¡£½ñÌìÓÚÙ»¸ºÔðÕÕ¹ËËû¡£
Ò»´óÔçÍõÃ÷¾ÍÈ¥ÕÒÓÚÙ»Ò»ÆðÉÏÑ§£¬Ëû¸Õ´ÓÒ»³¡´ó²¡ÖÐ»Ö¸´¹ýÀ´¡£½ñÌìÊÇÓÚÙ»¸ºÔðÕÕ¹ËËû¡£
Ò»´óÔçÍõÃ÷¾ÍÈ¥ÕÒÓÚÙ»Ò»ÆðÉÏÑ§£¬Ëû¸Õ´ÓÒ»³¡´ó²¡ÖÐ»Ö¸´¹ýÀ´¡£½ñÌìÓÚÙ»¸ºÔðÕÕ¹ËËû¡£
ÍõÃ÷¸Õ¸Õ´Ó¹úÍâ»ØÀ´¡£

11
ÕÅÉºÌÉÔÚ²ÝµØÉÏºÜ¿ìË¯×ÅÁË£¬ËýÃÎµ½ÃÀÀöµÄÌìÌÃºÍÈ¥ÊÀµÄÀÑÀÑ¡£ÕâÊ±ÊÇÐ»ÓîÇáÇáµØÒ¡ÐÑÁËËý¡£
ÕÅÉºÌÉÔÚ²ÝµØÉÏºÜ¿ìË¯×ÅÁË£¬ËýÃÎµ½ÃÀÀöµÄÌìÌÃºÍÈ¥ÊÀµÄÀÑÀÑ¡£ÕâÊ±Ð»ÓîÇáÇáµØÒ¡ÐÑÁËËý¡£
ÕÅÉºÔÚµÈÐ»Óî»ØÀ´µÄÊ±ºòË¯×ÅÁË£¬ËýÃÎµ½ÃÀÀöµÄÌìÌÃºÍÈ¥ÊÀµÄÀÑÀÑ¡£ÕâÊ±ÊÇÐ»ÓîÇáÇáµØÒ¡ÐÑÁËËý¡£
ÕÅÉºÔÚµÈÐ»Óî»ØÀ´µÄÊ±ºòË¯×ÅÁË£¬ËýÃÎµ½ÃÀÀöµÄÌìÌÃºÍÈ¥ÊÀµÄÀÑÀÑ¡£ÕâÊ±Ð»ÓîÇáÇáµØÒ¡ÐÑÁËËý¡£
ÕÅÉºÃÎµ½ÁËÈ¥ÊÀµÄÀÑÀÑ¡£

12
ÖÜÏÈÉú×î½üÔÚ³ï±¸Ò»±¾ÔÓÖ¾£¬ËûÕýÎªÔÓÖ¾µÄÃû×Ö¶ø¿àÄÕ¡£ºóÀ´ÊÇÓàÀöÎÞÒâÖÐÆô·¢ÁËËû¡£
ÖÜÏÈÉú×î½üÔÚ³ï±¸Ò»±¾ÔÓÖ¾£¬ËûÕýÎªÔÓÖ¾µÄÃû×Ö¶ø¿àÄÕ¡£ºóÀ´ÓàÀöÎÞÒâÖÐÆô·¢ÁËËû¡£
ÖÜÏÈÉú¸æËßÓàÀöËûÔÚ³ï±¸ÔÓÖ¾£¬ËûÕýÎªÔÓÖ¾µÄÃû×Ö¶ø¿àÄÕ¡£ºóÀ´ÊÇÓàÀöÎÞÒâÖÐÆô·¢ÁËËû¡£
ÖÜÏÈÉú¸æËßÓàÀöËûÔÚ³ï±¸ÔÓÖ¾£¬ËûÕýÎªÔÓÖ¾µÄÃû×Ö¶ø¿àÄÕ¡£ºóÀ´ÓàÀöÎÞÒâÖÐÆô·¢ÁËËû¡£

13
Àî½¨²Î¼ÓÁËÑ§Ð£ÀïµÄ¶¯Îï±£»¤Ð­»á£¬Ëû×î½üÔÚÑ§Ï°ÈçºÎÖÎÁÆÊÜÉËÀ¥³æ¡£Õâ¼¸ÌìÊÇÅíÝÕÊÖ°ÑÊÖµØ½ÌËû¡£
Àî½¨²Î¼ÓÁËÑ§Ð£ÀïµÄ¶¯Îï±£»¤Ð­»á£¬Ëû×î½üÔÚÑ§Ï°ÈçºÎÖÎÁÆÊÜÉËÀ¥³æ¡£Õâ¼¸ÌìÅíÝÕÊÖ°ÑÊÖµØ½ÌËû¡£
Àî½¨²Î¼ÓÁËÅíÝÕ·¢ÆðµÄ¶¯Îï±£»¤Ð­»á£¬Ëû×î½üÔÚÑ§Ï°ÈçºÎÖÎÁÆÊÜÉËÀ¥³æ¡£Õâ¼¸ÌìÊÇÅíÝÕÊÖ°ÑÊÖµØ½ÌËû¡£
Àî½¨²Î¼ÓÁËÅíÝÕ·¢ÆðµÄ¶¯Îï±£»¤Ð­»á£¬Ëû×î½üÔÚÑ§Ï°ÈçºÎÖÎÁÆÊÜÉËÀ¥³æ¡£Õâ¼¸ÌìÅíÝÕÊÖ°ÑÊÖµØ½ÌËû¡£
Àî½¨ÕýÔÚÑ§Ï°¾ÈÖÎÀ¥³æ¡£

14
½ã½ã½ñÌì¾ÍÒª¼Þ³öÈ¥ÁË£¬ËýÒ»´óÔç¾ÍÆðÀ´Êá×±´ò°ç¡£¹ýÁË»á¶ùÊÇÐí±òÃÀ×Ì×ÌµØÀ´½ÓËý¡£
½ã½ã½ñÌì¾ÍÒª¼Þ³öÈ¥ÁË£¬ËýÒ»´óÔç¾ÍÆðÀ´Êá×±´ò°ç¡£¹ýÁË»á¶ùÐí±òÃÀ×Ì×ÌµØÀ´½ÓËý¡£
½ã½ã½ñÌì¾ÍÒª¼Þ¸øÐí±òÁË£¬ËýÒ»´óÔç¾ÍÆðÀ´Êá×±´ò°ç¡£¹ýÁË»á¶ùÊÇÐí±òÃÀ×Ì×ÌµØÀ´½ÓËý¡£
½ã½ã½ñÌì¾ÍÒª¼Þ¸øÐí±òÁË£¬ËýÒ»´óÔç¾ÍÆðÀ´Êá×±´ò°ç¡£¹ýÁË»á¶ùÐí±òÃÀ×Ì×ÌµØÀ´½ÓËý¡£

15
ÂèÂè½ñÌìÃ¦µÃ²»¿É¿ª½»£¬ËýÉõÖÁ¶¼ÌÚ²»³öÊ±¼äÀ´Ï´ÒÂ·þ¡£Íí·¹ºóÊÇÒ¦ÎäÇÄÇÄµØ°ïÁËËý¡£
ÂèÂè½ñÌìÃ¦µÃ²»¿É¿ª½»£¬ËýÉõÖÁ¶¼ÌÚ²»³öÊ±¼äÀ´Ï´ÒÂ·þ¡£Íí·¹ºóÒ¦ÎäÇÄÇÄµØ°ïÁËËý¡£
ÂèÂè½ñÌìÃ¦µÃ¹Ë²»ÉÏÒ¦Îä£¬ËýÉõÖÁ¶¼ÌÚ²»³öÊ±¼äÀ´Ï´ÒÂ·þ¡£Íí·¹ºóÊÇÒ¦ÎäÇÄÇÄµØ°ïÁËËý¡£
ÂèÂè½ñÌìÃ¦µÃ¹Ë²»ÉÏÒ¦Îä£¬ËýÉõÖÁ¶¼ÌÚ²»³öÊ±¼äÀ´Ï´ÒÂ·þ¡£Íí·¹ºóÒ¦ÎäÇÄÇÄµØ°ïÁËËý¡£

16
»Æ²©ÏÂ°àºó¼±×Å¸Ï»Ø¼Ò£¬¹ýÈËÐÐºáµÀÊ±ËûÊÔÍ¼´³ºìµÆ¡£ÕâÊ±ºòÊÇÂÀÉ¯½÷É÷µØÖÆÖ¹ÁËËû¡£
»Æ²©ÏÂ°àºó¼±×Å¸Ï»Ø¼Ò£¬¹ýÈËÐÐºáµÀÊ±ËûÊÔÍ¼´³ºìµÆ¡£ÕâÊ±ºòÂÀÉ¯½÷É÷µØÖÆÖ¹ÁËËû¡£
»Æ²©ÏÂ°àºóË³µÀºÍÂÀÉ¯Ò»Æð»Ø¼Ò£¬¹ýÈËÐÐºáµÀÊ±ËûÊÔÍ¼´³ºìµÆ¡£ÕâÊ±ºòÊÇÂÀÉ¯½÷É÷µØÖÆÖ¹ÁËËû¡£
»Æ²©ÏÂ°àºóË³µÀºÍÂÀÉ¯Ò»Æð»Ø¼Ò£¬¹ýÈËÐÐºáµÀÊ±ËûÊÔÍ¼´³ºìµÆ¡£ÕâÊ±ºòÂÀÉ¯½÷É÷µØÖÆÖ¹ÁËËû¡£
»Æ²©´òËã´³ºìµÆ¡£

17
Àî»ªÔÚÂÛÌ³ÉÏ·¢ÁË¸öÌû×Ó£¬ËûËµ¸Ð¾õ»î×ÅÒ»µãÒâË¼¶¼Ã»ÓÐ¡£ºóÀ´ÊÇËÎ»ÝÄÍÐÄµØ¿ªµ¼ÁËËû¡£
Àî»ªÔÚÂÛÌ³ÉÏ·¢ÁË¸öÌû×Ó£¬ËûËµ¸Ð¾õ»î×ÅÒ»µãÒâË¼¶¼Ã»ÓÐ¡£ºóÀ´ËÎ»ÝÄÍÐÄµØ¿ªµ¼ÁËËû¡£
Àî»ªÔÚËÎ»ÝÈÎ°æÖ÷µÄÂÛÌ³ÉÏ·¢Ìû£¬ËûËµ¸Ð¾õ»î×ÅÒ»µãÒâË¼¶¼Ã»ÓÐ¡£ºóÀ´ÊÇËÎ»ÝÄÍÐÄµØ¿ªµ¼ÁËËû¡£
Àî»ªÔÚËÎ»ÝÈÎ°æÖ÷µÄÂÛÌ³ÉÏ·¢Ìû£¬ËûËµ¸Ð¾õ»î×ÅÒ»µãÒâË¼¶¼Ã»ÓÐ¡£ºóÀ´ËÎ»ÝÄÍÐÄµØ¿ªµ¼ÁËËû¡£

18
ºÎÃúÀ´µ½ÁËµÆ»ð»Ô»ÍµÄ´óÀñÌÃ£¬Ëû¿ªÊ¼¾Û¾«»áÉñµØ¿´½ÚÄ¿µ¥¡£Ô­À´ÊÇ·ëæÂÌØµØÑûÇëÁËËû¡£
ºÎÃúÀ´µ½ÁËµÆ»ð»Ô»ÍµÄ´óÀñÌÃ£¬Ëû¿ªÊ¼¾Û¾«»áÉñµØ¿´½ÚÄ¿µ¥¡£Ô­À´·ëæÂÌØµØÑûÇëÁËËû¡£
ºÎÃúÔØ×Å·ëæÂÀ´µ½´óÀñÌÃ£¬Ëû¿ªÊ¼¾Û¾«»áÉñµØ¿´½ÚÄ¿µ¥¡£Ô­À´ÊÇ·ëæÂÌØµØÑûÇëÁËËû¡£
ºÎÃúÔØ×Å·ëæÂÀ´µ½´óÀñÌÃ£¬Ëû¿ªÊ¼¾Û¾«»áÉñµØ¿´½ÚÄ¿µ¥¡£Ô­À´·ëæÂÌØµØÑûÇëÁËËû¡£
ºÎÃúµ½ÁËÀñÌÃºó¾Í×øÏÂ´òíï¡£

19
ÂíÏé´òËãºÍ´ó¼ÒÒ»ÆðÈ¥ÅÜÂíÀ­ËÉ£¬µ«Ëûµ£ÐÄ×Ô¼ºÅÜ²»ÁËÄÇÃ´Ô¶¡£ºóÀ´ÊÇ¶Åá°ÉîÉîµØ¹ÄÎèÁËËû¡£
ÂíÏé´òËãºÍ´ó¼ÒÒ»ÆðÈ¥ÅÜÂíÀ­ËÉ£¬µ«Ëûµ£ÐÄ×Ô¼ºÅÜ²»ÁËÄÇÃ´Ô¶¡£ºóÀ´¶Åá°ÉîÉîµØ¹ÄÎèÁËËû¡£
ÂíÏé´òËãºÍ¶Åá°ËýÃÇÈ¥ÅÜÂíÀ­ËÉ£¬µ«Ëûµ£ÐÄ×Ô¼ºÅÜ²»ÁËÄÇÃ´Ô¶¡£ºóÀ´ÊÇ¶Åá°ÉîÉîµØ¹ÄÎèÁËËû¡£
ÂíÏé´òËãºÍ¶Åá°ËýÃÇÈ¥ÅÜÂíÀ­ËÉ£¬µ«Ëûµ£ÐÄ×Ô¼ºÅÜ²»ÁËÄÇÃ´Ô¶¡£ºóÀ´¶Åá°ÉîÉîµØ¹ÄÎèÁËËû¡£

20
½ñÌìÐìÄÈºÍÍ¬Ñ§ÃÇÈ¥É­ÁÖ¹«Ô°Íæ£¬ËýÏÈ×·×Å»¨´ÔÖÐµÄºûµûÍæÁËÒ»»á¶ù¡£½Ó×ÅÊÇÈÎ·«ÍµÍµ°íµ¹ÁËËý¡£
½ñÌìÐìÄÈºÍÍ¬Ñ§ÃÇÈ¥É­ÁÖ¹«Ô°Íæ£¬ËýÏÈ×·×Å»¨´ÔÖÐµÄºûµûÍæÁËÒ»»á¶ù¡£½Ó×ÅÈÎ·«ÍµÍµ°íµ¹ÁËËý¡£
½ñÌìÐìÄÈºÍÈÎ·«ËûÃÇÈ¥É­ÁÖ¹«Ô°Íæ£¬ËýÏÈ×·×Å»¨´ÔÖÐµÄºûµûÍæÁËÒ»»á¶ù¡£½Ó×ÅÊÇÈÎ·«ÍµÍµ°íµ¹ÁËËý¡£
½ñÌìÐìÄÈºÍÈÎ·«ËûÃÇÈ¥É­ÁÖ¹«Ô°Íæ£¬ËýÏÈ×·×Å»¨´ÔÖÐµÄºûµûÍæÁËÒ»»á¶ù¡£½Ó×ÅÈÎ·«ÍµÍµ°íµ¹ÁËËý¡£

21
Îâ·¼½ñÌì´òÁË°ëÐ¡Ê±µÄÆ¹ÅÒÇò£¬Ëý¾õµÃ×Ô¼º½ø²½ºÜ´ó¡£Ô­À´ÊÇ½¯Ä¬ÈÏÕæµØÖ¸µ¼ÁËËý¡£
Îâ·¼½ñÌì´òÁË°ëÐ¡Ê±µÄÆ¹ÅÒÇò£¬Ëý¾õµÃ×Ô¼º½ø²½ºÜ´ó¡£Ô­À´½¯Ä¬ÈÏÕæµØÖ¸µ¼ÁËËý¡£
Îâ·¼ºÍ½¯Ä¬´òÁË°ëÐ¡Ê±Æ¹ÅÒÇò£¬Ëý¾õµÃ×Ô¼º½ø²½ºÜ´ó¡£Ô­À´ÊÇ½¯Ä¬ÈÏÕæµØÖ¸µ¼ÁËËý¡£
Îâ·¼ºÍ½¯Ä¬´òÁË°ëÐ¡Ê±Æ¹ÅÒÇò£¬Ëý¾õµÃ×Ô¼º½ø²½ºÜ´ó¡£Ô­À´½¯Ä¬ÈÏÕæµØÖ¸µ¼ÁËËý¡£
Îâ·¼½ñÌì´òÁË°ëÐ¡Ê±ÓðÃ«Çò¡£

22
Áõ·ÉÖÕÓÚ×öÍêÁËÖÜÄ©×÷Òµ£¬Ëû¿ªÊ¼¿´×îÏ²»¶µÄÐÇ¼ÊÃÔº½¡£ÕâÊ±ºòÊÇ³ÌÒð¼±´Ò´ÒµØÀ´ÕÒËû¡£
Áõ·ÉÖÕÓÚ×öÍêÁËÖÜÄ©×÷Òµ£¬Ëû¿ªÊ¼¿´×îÏ²»¶µÄÐÇ¼ÊÃÔº½¡£ÕâÊ±ºò³ÌÒð¼±´Ò´ÒµØÀ´ÕÒËû¡£
Áõ·É¸æ±ð³ÌÒðºó»Øµ½¼ÒÀï£¬Ëû¿ªÊ¼¿´×îÏ²»¶µÄÐÇ¼ÊÃÔº½¡£ÕâÊ±ºòÊÇ³ÌÒð¼±´Ò´ÒµØÀ´ÕÒËû¡£
Áõ·É¸æ±ð³ÌÒðºó»Øµ½¼ÒÀï£¬Ëû¿ªÊ¼¿´×îÏ²»¶µÄÐÇ¼ÊÃÔº½¡£ÕâÊ±ºò³ÌÒð¼±´Ò´ÒµØÀ´ÕÒËû¡£

23
ÍõÒã±»ËÍ½ø¸ô±ÚÄÇ¼Ò´óÒ½Ôº£¬ËûÔÚ¸ßËÙ¹«Â·ÉÏ³öÁË³µ»ö¡£ÌýËµºóÀ´ÊÇÁºÃ·Á¬Ò¹ÇÀ¾ÈÁËËû¡£
ÍõÒã±»ËÍ½ø¸ô±ÚÄÇ¼Ò´óÒ½Ôº£¬ËûÔÚ¸ßËÙ¹«Â·ÉÏ³öÁË³µ»ö¡£ÌýËµºóÀ´ÁºÃ·Á¬Ò¹ÇÀ¾ÈÁËËû¡£
ÍõÒã±»ËÍ½øÁºÃ·¹¤×÷µÄÒ½Ôº£¬ËûÔÚ¸ßËÙ¹«Â·ÉÏ³öÁË³µ»ö¡£ÌýËµºóÀ´ÊÇÁºÃ·Á¬Ò¹ÇÀ¾ÈÁËËû¡£
ÍõÒã±»ËÍ½øÁºÃ·¹¤×÷µÄÒ½Ôº£¬ËûÔÚ¸ßËÙ¹«Â·ÉÏ³öÁË³µ»ö¡£ÌýËµºóÀ´ÁºÃ·Á¬Ò¹ÇÀ¾ÈÁËËû¡£

24
ºÎÈÊÕýÔÚÈ°ËµÅóÓÑÃÇºÍËûÈ¥½¼ÓÎ£¬¸ù±¾²»¹ÜÌìÆøÔ¤±¨ËµÒª±äÌì¡£ÕâÊ±ºòÊÇÖÓÓ¨ÀíÖÇµØ·´¶ÔËû¡£
ºÎÈÊÕýÔÚÈ°ËµÅóÓÑÃÇºÍËûÈ¥½¼ÓÎ£¬¸ù±¾²»¹ÜÌìÆøÔ¤±¨ËµÒª±äÌì¡£ÕâÊ±ºòÖÓÓ¨ÀíÖÇµØ·´¶ÔËû¡£
ºÎÈÊÕýÔÚÈ°ËµÖÓÓ¨ËûÃÇÈ¥½¼ÓÎ£¬¸ù±¾²»¹ÜÌìÆøÔ¤±¨ËµÒª±äÌì¡£ÕâÊ±ºòÊÇÖÓÓ¨ÀíÖÇµØ·´¶ÔËû¡£
ºÎÈÊÕýÔÚÈ°ËµÖÓÓ¨ËûÃÇÈ¥½¼ÓÎ£¬¸ù±¾²»¹ÜÌìÆøÔ¤±¨ËµÒª±äÌì¡£ÕâÊ±ºòÖÓÓ¨ÀíÖÇµØ·´¶ÔËû¡£
ºÎÈÊÕýÔÚ¼Æ»®È¥½¼ÓÎ¡£

25
½ñÌìÕÅÐùÈ¥¸ç¸ç¼Ò´®ÃÅ£¬ËûºÜ¿ªÐÄµØÅãÐ¡Ö¶Å®ÇçÇçÍæ¡£Îç·¹Ê±ÊÇÖ£Âü¸ßÐËµØ¿ä½±ÁËËû¡£
½ñÌìÕÅÐùÈ¥¸ç¸ç¼Ò´®ÃÅ£¬ËûºÜ¿ªÐÄµØÅãÐ¡Ö¶Å®ÇçÇçÍæ¡£Îç·¹Ê±Ö£Âü¸ßÐËµØ¿ä½±ÁËËû¡£
½ñÌìÕÅÐùÈ¥É©×ÓÖ£Âü¼Ò´®ÃÅ£¬ËûºÜ¿ªÐÄµØÅãÐ¡Ö¶Å®ÇçÇçÍæ¡£Îç·¹Ê±ÊÇÖ£Âü¸ßÐËµØ¿ä½±ÁËËû¡£
½ñÌìÕÅÐùÈ¥É©×ÓÖ£Âü¼Ò´®ÃÅ£¬ËûºÜ¿ªÐÄµØÅãÐ¡Ö¶Å®ÇçÇçÍæ¡£Îç·¹Ê±Ö£Âü¸ßÐËµØ¿ä½±ÁËËû¡£

26
ÔøÈØµÃµ½×¨Ïî»ù½ðµÄ×ÊÖú£¬ËýÌá³öÒ»Ïî·çÏÕºÜ´óµÄ¼Æ»®¡£ÌýËµÊÇÑÖºê¼á¶¨µØÖ§³ÖÁËËý¡£
ÔøÈØµÃµ½×¨Ïî»ù½ðµÄ×ÊÖú£¬ËýÌá³öÒ»Ïî·çÏÕºÜ´óµÄ¼Æ»®¡£ÌýËµÑÖºê¼á¶¨µØÖ§³ÖÁËËý¡£
ÔøÈØµÃµ½ÑÖºê»ù½ðµÄ×ÊÖú£¬ËýÌá³öÒ»Ïî·çÏÕºÜ´óµÄ¼Æ»®¡£ÌýËµÊÇÑÖºê¼á¶¨µØÖ§³ÖÁËËý¡£
ÔøÈØµÃµ½ÑÖºê»ù½ðµÄ×ÊÖú£¬ËýÌá³öÒ»Ïî·çÏÕºÜ´óµÄ¼Æ»®¡£ÌýËµÑÖºê¼á¶¨µØÖ§³ÖÁËËý¡£

27
ÍõÜß×øÔÚ¿ªÍùÎ÷²ØµÄ»ð³µÉÏ£¬ËýÌÏÌÏ²»¾øµØÃèÊö×Ô¼ºµÄÏòÍù¡£ÕâÊ±ºòÊÇ¶¡ÕÜ²»ÄÍ·³µØ´ò¶ÏÁËËý¡£
ÍõÜß×øÔÚ¿ªÍùÎ÷²ØµÄ»ð³µÉÏ£¬ËýÌÏÌÏ²»¾øµØÃèÊö×Ô¼ºµÄÏòÍù¡£ÕâÊ±ºò¶¡ÕÜ²»ÄÍ·³µØ´ò¶ÏÁËËý¡£
ÍõÜßÓë¶¡ÕÜÉÏÁË¿ªÍùÎ÷²ØµÄ»ð³µ£¬ËýÌÏÌÏ²»¾øµØÃèÊö×Ô¼ºµÄÏòÍù¡£ÕâÊ±ºòÊÇ¶¡ÕÜ²»ÄÍ·³µØ´ò¶ÏÁËËý¡£
ÍõÜßÓë¶¡ÕÜÉÏÁË¿ªÍùÎ÷²ØµÄ»ð³µ£¬ËýÌÏÌÏ²»¾øµØÃèÊö×Ô¼ºµÄÏòÍù¡£ÕâÊ±ºò¶¡ÕÜ²»ÄÍ·³µØ´ò¶ÏÁËËý¡£

28
ÕÔÉôºÜµ£ÐÄ¹«Ë¾ÐÂÒ»ÂÖµÄ²ÃÔ±£¬ËýÕâÒ»¶ÎÊ±¼ä×Ü¾õµÃÐÄÉñ²»Äþ¡£ºóÀ´ÊÇº«ÓÂÄÍÐÄµØ°²Î¿ÁËËý¡£
ÕÔÉôºÜµ£ÐÄ¹«Ë¾ÐÂÒ»ÂÖµÄ²ÃÔ±£¬ËýÕâÒ»¶ÎÊ±¼ä×Ü¾õµÃÐÄÉñ²»Äþ¡£ºóÀ´º«ÓÂÄÍÐÄµØ°²Î¿ÁËËý¡£
ÕÔÉôºÜµ£ÐÄº«ÓÂÌá³öµÄ²ÃÔ±¼Æ»®£¬ËýÕâÒ»¶ÎÊ±¼ä×Ü¾õµÃÐÄÉñ²»Äþ¡£ºóÀ´ÊÇº«ÓÂÄÍÐÄµØ°²Î¿ÁËËý¡£
ÕÔÉôºÜµ£ÐÄº«ÓÂÌá³öµÄ²ÃÔ±¼Æ»®£¬ËýÕâÒ»¶ÎÊ±¼ä×Ü¾õµÃÐÄÉñ²»Äþ¡£ºóÀ´º«ÓÂÄÍÐÄµØ°²Î¿ÁËËý¡£

29
ÁÖºçÔ¶Ô¶µØ¿´µ½¸ßÖÐÄÇ°ïÍ¬Ñ§£¬ËýÐË·ÜµØÏòËûÃÇ»ÓÊÖ¡£ÄÇ±ßÊÇ´ÞÕñÊ×ÏÈ¿´µ½ÁËËý¡£
ÁÖºçÔ¶Ô¶µØ¿´µ½¸ßÖÐÄÇ°ïÍ¬Ñ§£¬ËýÐË·ÜµØÏòËûÃÇ»ÓÊÖ¡£ÄÇ±ß´ÞÕñÊ×ÏÈ¿´µ½ÁËËý¡£
ÁÖºçÔ¶Ô¶µØ¿´µ½ÁË´ÞÕñºÍºîÉÆ£¬ËýÐË·ÜµØÏòËûÃÇ»ÓÊÖ¡£ÄÇ±ßÊÇ´ÞÕñÊ×ÏÈ¿´µ½ÁËËý¡£
ÁÖºçÔ¶Ô¶µØ¿´µ½ÁË´ÞÕñºÍºîÉÆ£¬ËýÐË·ÜµØÏòËûÃÇ»ÓÊÖ¡£ÄÇ±ß´ÞÕñÊ×ÏÈ¿´µ½ÁËËý¡£

30
ÀÏÔ°¶¡Ò¯Ò¯ÔÚ»¨Ô°Àï×¨ÐÄÖÂÖ¾µØ¸É»î£¬ËûÕýÔÚÐÞ¼ôÒ»ÖêÄµµ¤µÄÖ¦Ò¶¡£ÕâÊ±ºòÊÇµËç÷ÃÍµØ×²µ¹ÁËËû¡£
ÀÏÔ°¶¡Ò¯Ò¯ÔÚ»¨Ô°Àï×¨ÐÄÖÂÖ¾µØ¸É»î£¬ËûÕýÔÚÐÞ¼ôÒ»ÖêÄµµ¤µÄÖ¦Ò¶¡£ÕâÊ±ºòµËç÷ÃÍµØ×²µ¹ÁËËû¡£
ÀÏÔ°¶¡Ò¯Ò¯ÔÚµËç÷¼ÒµÄ»¨Ô°Àï¹¤×÷£¬ËûÕýÔÚÐÞ¼ôÒ»ÖêÄµµ¤µÄÖ¦Ò¶¡£ÕâÊ±ºòÊÇµËç÷ÃÍµØ×²µ¹ÁËËû¡£
ÀÏÔ°¶¡Ò¯Ò¯ÔÚµËç÷¼ÒµÄ»¨Ô°Àï¹¤×÷£¬ËûÕýÔÚÐÞ¼ôÒ»ÖêÄµµ¤µÄÖ¦Ò¶¡£ÕâÊ±ºòµËç÷ÃÍµØ×²µ¹ÁËËû¡£

31
ÕÔ¸ÕÅª¶ªÁË¸ÕÄÃµ½²»¾ÃµÄÑÛ¾µ£¬Ëû¾ö¶¨ÔÙÈ¥ÅäÒ»¸±ÐÂµÄ¡£ºóÀ´ÊÇ½ªÈãÐÄÌÛµØÂñÔ¹Ëû¡£
ÕÔ¸ÕÅª¶ªÁË¸ÕÄÃµ½²»¾ÃµÄÑÛ¾µ£¬Ëû¾ö¶¨ÔÙÈ¥ÅäÒ»¸±ÐÂµÄ¡£ºóÀ´½ªÈãÐÄÌÛµØÂñÔ¹Ëû¡£
ÕÔ¸ÕÅª¶ªÁË½ªÈãËÍµÄÄÇ¸±ÑÛ¾µ£¬Ëû¾ö¶¨ÔÙÈ¥ÅäÒ»¸±ÐÂµÄ¡£ºóÀ´ÊÇ½ªÈãÐÄÌÛµØÂñÔ¹Ëû¡£
ÕÔ¸ÕÅª¶ªÁË½ªÈãËÍµÄÄÇ¸±ÑÛ¾µ£¬Ëû¾ö¶¨ÔÙÈ¥ÅäÒ»¸±ÐÂµÄ¡£ºóÀ´½ªÈãÐÄÌÛµØÂñÔ¹Ëû¡£

32
ÄÇÌìÕÅÃÀÔ­±¾ÒªÈ¥³¬ÊÐ¹ºÎï£¬Ëý¶¼ÒÑ¾­Ð´ºÃ¹ºÎïµ¥×¼±¸³öÃÅÁË¡£½á¹ûÊÇÐ¤»ÔÁÙÊ±½Ð×¡ÁËËý¡£
ÄÇÌìÕÅÃÀÔ­±¾ÒªÈ¥³¬ÊÐ¹ºÎï£¬Ëý¶¼ÒÑ¾­Ð´ºÃ¹ºÎïµ¥×¼±¸³öÃÅÁË¡£½á¹ûÐ¤»ÔÁÙÊ±½Ð×¡ÁËËý¡£
ÄÇÌìÕÅÃÀ¸æËßÐ¤»ÔÒªÈ¥ÌË³¬ÊÐ£¬Ëý¶¼ÒÑ¾­Ð´ºÃ¹ºÎïµ¥×¼±¸³öÃÅÁË¡£½á¹ûÊÇÐ¤»ÔÁÙÊ±½Ð×¡ÁËËý¡£
ÄÇÌìÕÅÃÀ¸æËßÐ¤»ÔÒªÈ¥ÌË³¬ÊÐ£¬Ëý¶¼ÒÑ¾­Ð´ºÃ¹ºÎïµ¥×¼±¸³öÃÅÁË¡£½á¹ûÐ¤»ÔÁÙÊ±½Ð×¡ÁËËý¡£

33
±í½ã½ñÌì´òËã³öÈ¥¹ä½Ö£¬ËýÔçÉÏÒ»ÆðÀ´¾Í¿ªÊ¼´òµç»°¡£°ëÐ¡Ê±ºóÊÇÐ¡ÑÕÐË³å³åµØÀ´½ÐËý¡£ 
±í½ã½ñÌì´òËã³öÈ¥¹ä½Ö£¬ËýÔçÉÏÒ»ÆðÀ´¾Í¿ªÊ¼´òµç»°¡£°ëÐ¡Ê±ºóÐ¡ÑÕÐË³å³åµØÀ´½ÐËý¡£
±í½ã½ñÌìÔ¼ÁËÐ¡ÑÕ¹ä½Ö£¬ËýÔçÉÏÒ»ÆðÀ´¾Í¿ªÊ¼´òµç»°¡£°ëÐ¡Ê±ºóÊÇÐ¡ÑÕÐË³å³åµØÀ´½ÐËý¡£
±í½ã½ñÌìÔ¼ÁËÐ¡ÑÕ¹ä½Ö£¬ËýÔçÉÏÒ»ÆðÀ´¾Í¿ªÊ¼´òµç»°¡£°ëÐ¡Ê±ºóÐ¡ÑÕÐË³å³åµØÀ´½ÐËý¡£
±í½ã½ñÌì´òËãÔÚÎÝÀï´òÉ¨ÎÀÉú¡£

34
Ð¡Éº×øÔÚ¿Î×ÀÇ°ÓÖ×ßÉñÁË£¬Ëý´ô´ôµØ¿´×Å´°Íâ²»ÖªµÀÔÚÏëÊ²Ã´¡£ÕâÊ±ÊÇÕÂËÉ×ß¹ýÀ´ÌáÐÑËý¡£
Ð¡ÉºÕýÔÚÈÏÕæµØÌý¿Î¡£
Ð¡Éº×øÔÚ¿Î×ÀÇ°ÓÖ×ßÉñÁË£¬Ëý´ô´ôµØ¿´×Å´°Íâ²»ÖªµÀÔÚÏëÊ²Ã´¡£ÕâÊ±ÕÂËÉ×ß¹ýÀ´ÌáÐÑËý¡£
Ð¡ÉºÔÚÕÂËÉÀÏÊ¦µÄ¿ÎÉÏÓÖ×ßÉñÁË£¬Ëý´ô´ôµØ¿´×Å´°Íâ²»ÖªµÀÔÚÏëÊ²Ã´¡£ÕâÊ±ÊÇÕÂËÉ×ß¹ýÀ´ÌáÐÑËý¡£
Ð¡ÉºÔÚÕÂËÉÀÏÊ¦µÄ¿ÎÉÏÓÖ×ßÉñÁË£¬Ëý´ô´ôµØ¿´×Å´°Íâ²»ÖªµÀÔÚÏëÊ²Ã´¡£ÕâÊ±ÕÂËÉ×ß¹ýÀ´ÌáÐÑËý¡£

35
ÄÌÄÌÒ»´óÔç¾ÍÉÏ²ËÊÐ³¡È¥ÁË£¬¿ìµ½ÖÐÎçÁË»¹Ã»ÓÐ»ØÀ´¡£ÕâÊ±ºòÊÇ°¢ÀÚ×Ô¸æ·ÜÓÂÇ°È¥ÕÒËý¡£
ÄÌÄÌÒ»´óÔç¾ÍÉÏ²ËÊÐ³¡È¥ÁË£¬¿ìµ½ÖÐÎçÁË»¹Ã»ÓÐ»ØÀ´¡£ÕâÊ±ºò°¢ÀÚ×Ô¸æ·ÜÓÂÇ°È¥ÕÒËý¡£
ÄÌÄÌ¶£ÖöÁË°¢ÀÚ¾ÍÉÏ²ËÊÐ³¡È¥ÁË£¬¿ìµ½ÖÐÎçÁË»¹Ã»ÓÐ»ØÀ´¡£ÕâÊ±ºòÊÇ°¢ÀÚ×Ô¸æ·ÜÓÂÇ°È¥ÕÒËý¡£
ÄÌÄÌ¶£ÖöÁË°¢ÀÚ¾ÍÉÏ²ËÊÐ³¡È¥ÁË£¬¿ìµ½ÖÐÎçÁË»¹Ã»ÓÐ»ØÀ´¡£ÕâÊ±ºò°¢ÀÚ×Ô¸æ·ÜÓÂÇ°È¥ÕÒËý¡£
ÄÌÄÌÉÏ²ËÊÐ³¡È¥ÁË¡£

36
Ç°¼¸ÌìÐ¡¹ùÏò¹«Ë¾ÉêÇëÔ¤Ö§Ò»ÄêÐ½Ë®£¬ÌýËµËû¼ÒÀï³öÁËµãÊÂ¡£µ±Ê±ÊÇÊæ½ãË¬¿ìµØÅú×¼ÁËËû¡£
Ç°¼¸ÌìÐ¡¹ùÏò¹«Ë¾ÉêÇëÔ¤Ö§Ò»ÄêÐ½Ë®£¬ÌýËµËû¼ÒÀï³öÁËµãÊÂ¡£µ±Ê±Êæ½ãË¬¿ìµØÅú×¼ÁËËû¡£
Ç°¼¸ÌìÐ¡¹ùÏòÊæ½ãÉêÇëÔ¤Ö§Ò»ÄêÐ½Ë®£¬ÌýËµËû¼ÒÀï³öÁËµãÊÂ¡£µ±Ê±ÊÇÊæ½ãË¬¿ìµØÅú×¼ÁËËû¡£
Ç°¼¸ÌìÐ¡¹ùÏòÊæ½ãÉêÇëÔ¤Ö§Ò»ÄêÐ½Ë®£¬ÌýËµËû¼ÒÀï³öÁËµãÊÂ¡£µ±Ê±Êæ½ãË¬¿ìµØÅú×¼ÁËËû¡£
Ð¡¹ùÔ¤Ö§ÁËÒ»ÄêÐ½Ë®¡£

37
Ð¡×£ÄÇÌìÍ»È»´Çµô¹¤×÷£¬»ØÀ´ºó¾ÍÊÕÊ°ÐÐÀîÀë¿ªÁËÎÚ½ðÐ¡Õò¡£ÈËÃÇ¶¼ËµÊÇÅÓÏÜ°µµØÀïÍþÐ²ÁËËý¡£
Ð¡×£ÄÇÌìÍ»È»´Çµô¹¤×÷£¬»ØÀ´ºó¾ÍÊÕÊ°ÐÐÀîÀë¿ªÁËÎÚ½ðÐ¡Õò¡£ÈËÃÇ¶¼ËµÅÓÏÜ°µµØÀïÍþÐ²ÁËËý¡£
Ð¡×£ÄÇÌì±»ÅÓÏÜ½Ð×ß£¬»ØÀ´ºó¾ÍÊÕÊ°ÐÐÀîÀë¿ªÁËÎÚ½ðÐ¡Õò¡£ÈËÃÇ¶¼ËµÊÇÅÓÏÜ°µµØÀïÍþÐ²ÁËËý¡£
Ð¡×£ÄÇÌì±»ÅÓÏÜ½Ð×ß£¬»ØÀ´ºó¾ÍÊÕÊ°ÐÐÀîÀë¿ªÁËÎÚ½ðÐ¡Õò¡£ÈËÃÇ¶¼ËµÅÓÏÜ°µµØÀïÍþÐ²ÁËËý¡£

38
ÕâÊ±ºòÏþÑ©ÐÄÈçµ¶¸î£¬ËýºÜÏëÕÒ¸öÃ»ÈËµÄµØ·½Í´¿ÞÒ»³¡¡£Ã»Ïëµ½ÊÇÁøºÆ±³µØÀï³öÂôÁËËý¡£
ÕâÊ±ºòÏþÑ©ÐÄÈçµ¶¸î£¬ËýºÜÏëÕÒ¸öÃ»ÈËµÄµØ·½Í´¿ÞÒ»³¡¡£Ã»Ïëµ½ÁøºÆ±³µØÀï³öÂôÁËËý¡£
ÏþÑ©ÄÑÒÔÖÃÐÅµØÍû×ÅÁøºÆ£¬ËýºÜÏëÕÒ¸öÃ»ÈËµÄµØ·½Í´¿ÞÒ»³¡¡£Ã»Ïëµ½ÊÇÁøºÆ±³µØÀï³öÂôÁËËý¡£
ÏþÑ©ÄÑÒÔÖÃÐÅµØÍû×ÅÁøºÆ£¬ËýºÜÏëÕÒ¸öÃ»ÈËµÄµØ·½Í´¿ÞÒ»³¡¡£Ã»Ïëµ½ÁøºÆ±³µØÀï³öÂôÁËËý¡£

39
ºúÀÏ°åÏò´ó¼ÒÕ¹Ê¾ÁË×Ô¼ºµÄ°®³µ£¬Ëû¶Ô°®³µµÄÐÔÄÜ·Ç³£ÂúÒâ¡£ºóÀ´ÊÇÐÏÒåÒ»Á³²»Ð¼µØ³°Ð¦ÁËËû¡£
ºúÀÏ°åÏò´ó¼ÒÕ¹Ê¾ÁË×Ô¼ºµÄ°®³µ£¬Ëû¶Ô°®³µµÄÐÔÄÜ·Ç³£ÂúÒâ¡£ºóÀ´ÐÏÒåÒ»Á³²»Ð¼µØ³°Ð¦ÁËËû¡£
ºúÀÏ°åÏòÐÏÒåÕ¹Ê¾ÁË×Ô¼ºµÄ°®³µ£¬Ëû¶Ô°®³µµÄÐÔÄÜ·Ç³£ÂúÒâ¡£ºóÀ´ÊÇÐÏÒåÒ»Á³²»Ð¼µØ³°Ð¦ÁËËû¡£
ºúÀÏ°åÏòÐÏÒåÕ¹Ê¾ÁË×Ô¼ºµÄ°®³µ£¬Ëû¶Ô°®³µµÄÐÔÄÜ·Ç³£ÂúÒâ¡£ºóÀ´ÐÏÒåÒ»Á³²»Ð¼µØ³°Ð¦ÁËËû¡£

40
ËïÀÏÊ¦´øº¢×ÓÃÇÈ¥²Î¹Û¶¯Îï²©Îï¹Ý£¬ËûÕýÔÚ¸ø´ó¼ÒÇ¿µ÷²©Îï¹Ý¹æ¶¨£¬ÕâÊ±ÊÇÏþÂ¶ÐË·ÜµØ´ò¶ÏÁËËû¡£
ËïÀÏÊ¦´øº¢×ÓÃÇÈ¥²Î¹Û¶¯Îï²©Îï¹Ý£¬ËûÕýÔÚ¸ø´ó¼ÒÇ¿µ÷²©Îï¹Ý¹æ¶¨£¬ÕâÊ±ÏþÂ¶ÐË·ÜµØ´ò¶ÏÁËËû¡£
ËïÀÏÊ¦´øÏþÂ¶ËýÃÇÈ¥²Î¹Û¶¯Îï²©Îï¹Ý£¬ËûÕýÔÚ¸ø´ó¼ÒÇ¿µ÷²©Îï¹Ý¹æ¶¨£¬ÕâÊ±ÊÇÏþÂ¶ÐË·ÜµØ´ò¶ÏÁËËû¡£
ËïÀÏÊ¦´øÏþÂ¶ËýÃÇÈ¥²Î¹Û¶¯Îï²©Îï¹Ý£¬ËûÕýÔÚ¸ø´ó¼ÒÇ¿µ÷²©Îï¹Ý¹æ¶¨£¬ÕâÊ±ÏþÂ¶ÐË·ÜµØ´ò¶ÏÁËËû¡£

41
Ð¡ÀîÊî¼Ùµ½Ò»¼Ò»á¼ÆÊ¦ÊÂÎñËùÊµÏ°£¬Ëû¸úÁËÒ»¸öÄÑ¶ÈºÜ´óµÄÉó¼ÆÏîÄ¿¡£ÄÇÊ±ºòÊÇ¸ðÁÕ²»Ê±µØÖ¸µãÏÂËû¡£
Ð¡ÀîÊî¼Ùµ½Ò»¼Ò»á¼ÆÊ¦ÊÂÎñËùÊµÏ°£¬Ëû¸úÁËÒ»¸öÄÑ¶ÈºÜ´óµÄÉó¼ÆÏîÄ¿¡£ÄÇÊ±ºò¸ðÁÕ²»Ê±µØÖ¸µãÏÂËû¡£
Ð¡ÀîÊî¼Ùµ½¸ðÁÕµÄ»á¼ÆÊ¦ÊÂÎñËùÊµÏ°£¬Ëû¸úÁËÒ»¸öÄÑ¶ÈºÜ´óµÄÉó¼ÆÏîÄ¿¡£ÄÇÊ±ºòÊÇ¸ðÁÕ²»Ê±µØÖ¸µãÏÂËû¡£
Ð¡ÀîÊî¼Ùµ½¸ðÁÕµÄ»á¼ÆÊ¦ÊÂÎñËùÊµÏ°£¬Ëû¸úÁËÒ»¸öÄÑ¶ÈºÜ´óµÄÉó¼ÆÏîÄ¿¡£ÄÇÊ±ºò¸ðÁÕ²»Ê±µØÖ¸µãÏÂËû¡£

42
½ñÌìÐ¡ÕÅÈ¥ÅóÓÑµÄ²è¹ÝÍæ£¬ÉúÒâÃ¦µÄÊ±ºòËû»¹¿Í´®Ò»ÏÂµêÐ¡¶þ¡£´òìÈÊ±ÊÇÄß½ãÕæ³ÏµØ¸ÐÐ»ÁËËû¡£
½ñÌìÐ¡ÕÅÈ¥ÅóÓÑµÄ²è¹ÝÍæ£¬ÉúÒâÃ¦µÄÊ±ºòËû»¹¿Í´®Ò»ÏÂµêÐ¡¶þ¡£´òìÈÊ±Äß½ãÕæ³ÏµØ¸ÐÐ»ÁËËû¡£
½ñÌìÐ¡ÕÅÈ¥Äß½ãµÄ²è¹ÝÍæ£¬ÉúÒâÃ¦µÄÊ±ºòËû»¹¿Í´®Ò»ÏÂµêÐ¡¶þ¡£´òìÈÊ±ÊÇÄß½ãÕæ³ÏµØ¸ÐÐ»ÁËËû¡£
½ñÌìÐ¡ÕÅÈ¥Äß½ãµÄ²è¹ÝÍæ£¬ÉúÒâÃ¦µÄÊ±ºòËû»¹¿Í´®Ò»ÏÂµêÐ¡¶þ¡£´òìÈÊ±Äß½ãÕæ³ÏµØ¸ÐÐ»ÁËËû¡£

43
ÕÔÃÃÃÃÍæÓÎÏ·Êä²»Æð£¬Ã¿´ÎÊäÁËËý¶¼Ë£Àµ²»ÈÏÕË¡£ºóÀ´ÊÇ¿ÂÐÇÆø·ßµØÖ¸ÔðÁËËý¡£
ÕÔÃÃÃÃÍæÓÎÏ·Êä²»Æð£¬Ã¿´ÎÊäÁËËý¶¼Ë£Àµ²»ÈÏÕË¡£ºóÀ´¿ÂÐÇÆø·ßµØÖ¸ÔðÁËËý¡£
ÕÔÃÃÃÃ¸ú¿ÂÐÇÍæÓÎÏ·Êä²»Æð£¬Ã¿´ÎÊäÁËËý¶¼Ë£Àµ²»ÈÏÕË¡£ºóÀ´ÊÇ¿ÂÐÇÆø·ßµØÖ¸ÔðÁËËý¡£
ÕÔÃÃÃÃ¸ú¿ÂÐÇÍæÓÎÏ·Êä²»Æð£¬Ã¿´ÎÊäÁËËý¶¼Ë£Àµ²»ÈÏÕË¡£ºóÀ´¿ÂÐÇÆø·ßµØÖ¸ÔðÁËËý¡£

44
Ïþ¼Ñ×Ô¼ºÒ»¸öÈËÀ´µ½»¬Ñ©³¡£¬ ´Ó½ñÌìÆðËýÒª¶ÀÁ¢½øÐÐ»¬Ñ©ÑµÁ·ÁË¡£¹ýÈ¥ÊÇÊ©ÑôÒ»Ö±Åã°é×ÅËý¡£
Ïþ¼Ñ×Ô¼ºÒ»¸öÈËÀ´µ½»¬Ñ©³¡£¬ ´Ó½ñÌìÆðËýÒª¶ÀÁ¢½øÐÐ»¬Ñ©ÑµÁ·ÁË¡£¹ýÈ¥Ê©ÑôÒ»Ö±Åã°é×ÅËý¡£ 
Ïþ¼Ñ¸æ±ðÊ©ÑôÀ´µ½»¬Ñ©³¡£¬ ´Ó½ñÌìÆðËýÒª¶ÀÁ¢½øÐÐ»¬Ñ©ÑµÁ·ÁË¡£¹ýÈ¥ÊÇÊ©ÑôÒ»Ö±Åã°é×ÅËý¡£
Ïþ¼Ñ¸æ±ðÊ©ÑôÀ´µ½»¬Ñ©³¡£¬ ´Ó½ñÌìÆðËýÒª¶ÀÁ¢½øÐÐ»¬Ñ©ÑµÁ·ÁË¡£¹ýÈ¥Ê©ÑôÒ»Ö±Åã°é×ÅËý¡£ 

45
ºÎÊåÊåÀ´µ½ÁËÓÎÀÖÔ°£¬ËûÒ»½øÃÅ¾ÍÏÈÈ¥ÂòÁË´®±ùÌÇºùÂ«¡£Ô­À´ÊÇÄÝÄÝ²»Í£µØ²ø×ÅËûÒª¡£
ºÎÊåÊåÀ´µ½ÁËÓÎÀÖÔ°£¬ËûÒ»½øÃÅ¾ÍÏÈÈ¥ÂòÁË´®±ùÌÇºùÂ«¡£Ô­À´ÄÝÄÝ²»Í£µØ²ø×ÅËûÒª¡£
ºÎÊåÊå´ø×ÅÄÝÄÝÀ´µ½ÓÎÀÖÔ°£¬ËûÒ»½øÃÅ¾ÍÏÈÈ¥ÂòÁË´®±ùÌÇºùÂ«¡£Ô­À´ÊÇÄÝÄÝ²»Í£µØ²ø×ÅËûÒª¡£
ºÎÊåÊå´ø×ÅÄÝÄÝÀ´µ½ÓÎÀÖÔ°£¬ËûÒ»½øÃÅ¾ÍÏÈÈ¥ÂòÁË´®±ùÌÇºùÂ«¡£Ô­À´ÄÝÄÝ²»Í£µØ²ø×ÅËûÒª¡£

46
ÍõÏþÈº±»¹¤×÷ÈËÔ±ÇëÉÏÁËÖ÷Ï¯Ì¨£¬ËûÈÙ»ñÄê¶È×î¼ÑÒµ¼¨½±¡£ÌýËµÊÇÄô×ÜÇ××ÔÎªËû°äÁË½±¡£
ÍõÏþÈº±»¹¤×÷ÈËÔ±ÇëÉÏÁËÖ÷Ï¯Ì¨£¬ËûÈÙ»ñÄê¶È×î¼ÑÒµ¼¨½±¡£ÌýËµÄô×ÜÇ××ÔÎªËû°äÁË½±¡£
ÍõÏþÈº±»Äô×ÜÇëÉÏÁËÖ÷Ï¯Ì¨£¬ËûÈÙ»ñÄê¶È×î¼ÑÒµ¼¨½±¡£ÌýËµÊÇÄô×ÜÇ××ÔÎªËû°äÁË½±¡£
ÍõÏþÈº±»Äô×ÜÇëÉÏÁËÖ÷Ï¯Ì¨£¬ËûÈÙ»ñÄê¶È×î¼ÑÒµ¼¨½±¡£ÌýËµÄô×ÜÇ××ÔÎªËû°äÁË½±¡£

47
Ñà×Ó¾­³£µ½ÐÄÖ®ÇÅÊéµêÂòÊé£¬½ñÌìËýÒª¸¶ÕÊÊ±²Å·¢ÏÖÇ®°üÍü´øÁË¡£ºóÀ´ÊÇÂ³³¬ÈÃËýÏÈÇ·×Å¡£
Ñà×Ó¾­³£µ½ÐÄÖ®ÇÅÊéµêÂòÊé£¬½ñÌìËýÒª¸¶ÕÊÊ±²Å·¢ÏÖÇ®°üÍü´øÁË¡£ºóÀ´Â³³¬ÈÃËýÏÈÇ·×Å¡£
Ñà×Ó¾­³£µ½Â³³¬µÄÊéµêÂòÊé£¬½ñÌìËýÒª¸¶ÕÊÊ±²Å·¢ÏÖÇ®°üÍü´øÁË¡£ºóÀ´ÊÇÂ³³¬ÈÃËýÏÈÇ·×Å¡£
Ñà×Ó¾­³£µ½Â³³¬µÄÊéµêÂòÊé£¬½ñÌìËýÒª¸¶ÕÊÊ±²Å·¢ÏÖÇ®°üÍü´øÁË¡£ºóÀ´Â³³¬ÈÃËýÏÈÇ·×Å¡£

48
»ÆÊ¦¸µ½ñÌìÍíÉÏÉúÒâºÜºÃ£¬ËûÒ»Ö±Ã¦µ½ÁË´ó°ëÒ¹²ÅÊÕÌ¯»Ø¼Ò¡£Àë¿ªÊ±ÊÇÀ¼ÃÃ¸Ï¹ýÀ´°ïËûÍÆ³µ¡£ 
»ÆÊ¦¸µ½ñÌìÍíÉÏÉúÒâºÜºÃ£¬ËûÒ»Ö±Ã¦µ½ÁË´ó°ëÒ¹²ÅÊÕÌ¯»Ø¼Ò¡£Àë¿ªÊ±À¼ÃÃ¸Ï¹ýÀ´°ïËûÍÆ³µ¡£
»ÆÊ¦¸µÈÃÀ¼ÃÃÏÈ»ØÈ¥³ÔÍí·¹£¬ËûÒ»Ö±Ã¦µ½ÁË´ó°ëÒ¹²ÅÊÕÌ¯»Ø¼Ò¡£Àë¿ªÊ±ÊÇÀ¼ÃÃ¸Ï¹ýÀ´°ïËûÍÆ³µ¡£
»ÆÊ¦¸µÈÃÀ¼ÃÃÏÈ»ØÈ¥³ÔÍí·¹£¬ËûÒ»Ö±Ã¦µ½ÁË´ó°ëÒ¹²ÅÊÕÌ¯»Ø¼Ò¡£Àë¿ªÊ±À¼ÃÃ¸Ï¹ýÀ´°ïËûÍÆ³µ¡£


Fillers
(Each item is one passage. The sentence after the passage, if any, is the comprehension question.)

Items 1
ÌýËµÊÇÌ·½ÜÔÚËµÏàÉùµÄÊ±ºòÍü´ÊÁË£¬»¹ºÃËûËæ»úÓ¦±ä°Ñ»°Ìâ²í¿ªÁË½Ó×Å½²¡£³ý´ËÖ®ÍâÆäËûÈË¶¼Ã»ÓÐÊ²Ã´ÒâÍâ¡£

2
ÊÇÍôÄÏ¸øÀîö©ÉÓÁË¸öÐÅ¶ù£¬Àîö©²ÅÖªµÀÄ¸Ç×Ç§ÀïÌöÌö¹ýÀ´ÁË¡£ËýºÞ²»µÃÂíÉÏÏÂ°à»Øµ½¼ÒÖÐ¡£
Àîö©µÄÄ¸Ç×¹ýÀ´ÁË¡£

3
ÊÇåû½ã½ñÌìÒªÈ¥ÅÄ»éÉ´ÕÕ£¬Ð¡Àò»¹µÃ¹ýÁ½Ìì²ÅÄÜÈ¥¡£×î½ü¹«Ë¾Ã¦£¬´ó¼ÒÖ»ÄÜÂÖÁ÷Çë¼Ù¡£

4
ÊÇ¸¶·å°ïÖÜ²®²®ÊÕ¸îµÄË®µ¾¡£ÖÜ²®²®µÄÐ¡Å©³¡½ñÄêÓÖÈ¡µÃ´ó·áÊÕ£¬Ëû×Ô¼ºÒ»¸öÈË»¹ÕæÊÇÃ¦²»¹ýÀ´ÁË¡£
ÖÜ²®²®µÄÐ¡Å©³¡½ñÄêÊÕ³É²»ºÃ¡£

5
ÌýËµÊÇ·½³ÙÈÇÉÏÁËÂé·³ÊÂ¡£ÄÑ¹Ö·½ÂèÂè×î½ü¼¸Ìì³îÃ¼²»Õ¹µÄ£¬Á¬Æ½Ê±×îÏ²»¶µÄÆåÅÆ¶¼²»ÍæÁË¡£

6
ÊÇÖÜ´ä×Ô¸ö¶ùÈ¥½»µÄË®µç·Ñ¡£±¾À´ËýÏëÈÃ×ÞÃñÉÏ°àÊ±Ë³Â·½»ÁË£¬½á¹ûÁ¬Ðø¼¸ÌìËû¶¼¸øÍüÁË¡£

7
Õâ¼þÊÂÎÒÃÇ²»ÄÜ¹ÖÍõð©¡£Ê×ÏÈÊÇÃÏÃØÊéÍ¨Öª´íÁË»áÒéµØµã£¬´ó¼ÒÓÖ¶¼´ÖÐÄÃ»ÓÐ½øÒ»²½ºË¶Ô¡£
ÃÏÃØÊéÍ¨Öª´íÁË»áÒéÊ±¼ä¡£

8
Àî½øÑûÇë´ó»ï¶ùÈ¥¿´ËûÖ÷³ÖµÄµÚÒ»³¡ÑÝ³ö¡£ºóÀ´ÊÇÇØËªÃ°Ã°Ê§Ê§µØ´òÁË¸ö²í£¬Àî½øÒ»½ôÕÅ¾ÍÍü´ÊÁË¡£

9
¶ÎºÀÕýÔÚÌÉÒÎÉÏÓÆÏÐµØÏíÊÜ¼ÙÈÕ¡£ÕâÊ±ºòÊÇ¼ÖÇí¹ýÀ´ÇëËû°ïÃ¦£¬´òÈÅÁËËûÄÑµÃÇåÏÐµÄÏÂÎç¡£

10
³Â×Ü°ÑãÆÆ¼½ÐÈ¥ÅúÁËÒ»¶Ù¡£×î½üÊÇãÆÆ¼¸ºÔð°²ÅÅËûµÄÐÐ³Ì£¬×òÌìËý°ÑÒ»¸öÖØÒª»áÒéµÄÊ±¼ä¸ã´íÁË¡£
ãÆÆ¼±»ÅúÆÀÁËÒ»¶Ù¡£

11
ÑîÖÇ´ø¶Î¶ðÈ¥²Î¹ÛÒ»¸öÅóÓÑµÄ»­Õ¹¡£ÔÚÕ¹ÌüÀïÊÇ¶Î¶ðÏòËû×öÁË×¨ÒµµÄ½²½â£¬Ô­À´ËýÔø¾­Ñ§¹ýÒ»¶ÎÊ±¼äµÄ»æ»­¡£

12
ÕýÔÂ³õÈýÕÔ¶«ËÍºîæ¿»ØÄï¼Ò¡£°ëÂ·ÉÏÊÇºîæ¿ÏëÆðÍü´øÄê»õÁË£¬ËýÈÃÕÔ¶«Æï³µ»ØÈ¥È¡¡£
ÕýÔÂ³õÈýºîæ¿»ØÄï¼Ò¡£

13
ÕÅÑÅÒ»ÈËÔÚ¼Ò½øÐÐ´óÉ¨³ý¡£ÂúÎÝ×Ó»Ò³¾µÄÊ±ºòÕÉ·ò»ØÀ´ÁË£¬ÕÅÑÅÈÃËûÏÈµ½ÍâÃæÁï´ïÒ»»á¶ù¡£

14
ÁõÁ«ºÍÅóÓÑÃÇÍíÉÏÈ¥ÁË¾Æ°É¡£ËýÃÇÇáËÉã«ÒâµØºÈ¾ÆÏÐÁÄ£¬Ëý¸Ðµ½½ôÕÅµÄÉñ¾­ÖÕÓÚËÉ³ÚÏÂÀ´¡£
ÁõÁ«ËýÃÇÈ¥ÁË²è¹Ý¡£

15
³ÂÀòÈ¥²Î¼ÓÒ»ÃÅÖØÒªµÄ¿¼ÊÔ£¬µ½ÁË¿¼³¡È´·¢ÏÖ×¼¿¼Ö¤ÍüÄÃÁË¡£»¹ºÃÒüæÃÓÐ¿Õ¿ÉÒÔ°ïËý´ø¹ýÀ´¡£

16
Ã«¶°ÌáÒéÏÂ°àÖ®ºó´ó¼ÒÈ¥K¸è¡£Á¬×î²»Ï²»¶³ª¸èµÄÐ¡Àî¶¼Í¬ÒâÁË¡£²»ÁÏñûÕêÌá³öÁË·´¶ÔÒâ¼û¡£
Ã«¶°ÌáÒéÏÂ°àºóÈ¥K¸è¡£

17
Ç®Î°ºÍÉÛÄÝÔÚÌÔ±¦¿ªÁËÒ»¸öÐ¡µê¡£ËûÃÇÏë¾¡¿ìÌáÉýÐ¡µêµÄÈËÆø£¬Îª´ËÇ®Î°³¢ÊÔÁËÎÞÊý°ì·¨¡£

18
Ð¡ÁÖ×î½ü³ÕÃÔÓÚ¸ãÐ¡·¢Ã÷¡£×òÌìËû¸Õ·¢Ã÷ÁËÒ»ÖÖ¶à¹¦ÄÜ»·±£´ü¡£½ñÌìÄªÇí¸æËßËû¼¸ÄêÇ°¾ÍÓÐÕâ¸ö·¢Ã÷ÁË¡£
¶à¹¦ÄÜ»·±£´üÊÇ×îÐÂµÄ·¢Ã÷¡£

19
ÌýËµÐÜÝæÍ¨¹ýÁË×îºóµÄÃæÊÔ¡£µ«ÊÇºóÀ´ÒòÎªË«·½¹ÛµãµÄ·ÖÆç£¬Ëý¿¼ÂÇÔÙÈý¾ö¶¨·ÅÆúÕâ¸ö»ú»á¡£

20
´ó¼ÒÊÇÔÚµãÃûÊ±·¢ÏÖ¼ÖÍþ²»¼ûÁËµÄ¡£ÉÏ¿ÎÇ°ºÜ¶àÈË¼ûµ½Ëû½øÀ´¹ý£¬µ«Ã»ÓÐÈË×¢Òâµ½ËûÉ¶Ê±ºò³öÈ¥ÁË¡£

21
ÑîÖ¥Õý°ÙÎÞÁÄÀµµØÌý×ÅÏÄÖÛµÄ¿Î£¬Í»È»ÏÄÖÛµÄÒ»¾ä»°ÒýÆðÁËËýµÄ×¢Òâ¡£ËûËµÈËÆ½¾ùÖ»Ðè7·ÖÖÓ¾Í¿ÉÒÔÈëË¯¡£

22
ÃÏ±õÔÚº£Ì²ÉÏÅöµ½Ò»¸öÐ¡Å®º¢½ÐÌ·Ö¥¡£Ì·Ö¥¸æËßËû×Ô¼ºÊÇÒ»¸ö¹Â¶ù£¬ÆäÊµÕâÊÇÌ·Ö¥±àµÄÒ»¸ö»Ñ»°¡£
Ì·Ö¥ÊÇÒ»¸ö¹Â¶ù¡£

23
½ðçùÏò°àÖ÷ÈÎÒü±øÇë¼ÙÈýÌì£¬ËýÒª»Ø¼ÒÈ¥±¼Ì«ÀÑÀÑµÄÉ¥¡£Òü±øÍ¨Çé´ïÀíµØÅú×¼ÁË¡£
½ðçùÇë¼Ù»ØÈ¥²Î¼ÓÌ«ÀÑÀÑµÄÔáÀñ¡£

24
ÀèÃô²ø×ÅÒª¸ú°Ö°Ö³öº£²¶Óã¡£È«¼ÒÈË¶¼¶ÔÕâÐ¡¹íºÁÎÞ°ì·¨£¬ºóÀ´Ò¯Ò¯ÓÃ¿Ö²ÀµÄöèÓãÏÅ×¡ÁËËý¡£
Ò¯Ò¯ÓÃöèÓãÕò×¡ÁËËý¡£

25
ÄÇ´ÎÊÇ°×Ô¾Ææ¼£°ã³öÏÖ¾ÈÁËÎâÝº¡£·ñÔòËýÒ»¸öÈË¸ù±¾ÎÞ·¨¶Ô¿¹ÄÇÖ»²Øéá£¬»á³öÏÖÔõÑùµÄ½á¹ûÖ»ÄÜÌýÌìÓÉÃüÁË¡£

26
ÊÇ¹ùÌ«Ì«¾ö¶¨°Ñ¼ÒÀïµÄÏÐÇ®Í¶×Êµ½¹ÉÆ±ÉÏ¡£µ±³õ¹ùÏÈÉúºÍËýÉÌÁ¿µÄÊ±ºò£¬Ëý¾Í·Ç³£ÀíÐÔµØ·ÖÎöÁË¸÷ÖÖ·½°¸µÄÓÅÁÓ¡£
¼ÒÀïµÄÏÐÇ®¶¼Í¶×Êµ½¹úÕ®ÉÏÁË¡£

27
ÌýËµÊÇÖìÍ®×¨³ÌÈ¥½ÓµÄºúÀû¡£ËûµÄÊ±¼ä»úÆ÷³öÁËµã¹ÊÕÏ£¬Í£ÁôÔÚ¹«Ôª1894Äê»Ø²»À´ÁË¡£

28
Õâ´Î¿¼ÊÔÊÇÌÕ¹¦ÄÃÁËµÚÒ»¡£ÒòÎªÕâ´ÎÎïÀí¿¼Ìâ³öÆæµÄÄÑ£¬¶øÎïÀíÇ¡ÊÇËûµÄÇ¿Ïî¡£

29
ÌÀÇ¿Ò»Ö±ÒÔÎªÊÇÏòæµ×¼±¸µÄÉúÈÕÍí»á¡£Ö±µ½ÓÐÒ»ÌìËûÎÞÒâÖÐ·¢ÏÖÁËÕæÏà£¬Ô­À´ÕâÒ»ÇÐ¶¼ÊÇÂèÂèÄ»ºó²ß»®µÄ¡£
ÊÇÂèÂè×¼±¸µÄÉúÈÕÍí»á¡£

30
¾ÝËµÊÇ¸µÊ«µÁÇÔÁËÄÇ¿ÅÏ¡ÊÀÃ÷Öé¡£²»¹ýÕâÖ»ÊÇÃñ¼äµÄ´«ÎÅºÍÍÆ²â£¬¹Ù·½¸ù±¾ÕÒ²»µ½Ò»Ë¿Ò»ºÁµÄÖ¤¾ÝÀ´¡£

31
ÄÇÒ»Äê¶¬ÌìÏÂÁËºÜ´óµÄÒ»³¡Ñ©£¬ÔÚ°×Ã£Ã£µÄ´óµØÉÏÊÇºÂÜ¿Ð¦ØÌÈç»¨£¬ÎÂÅ¯ÁËÕâÆ¬ÇåÀä¶øËØ½àµÄ´óµØ¡£

32
ÂÞ¹ãµÄ¹«Ë¾ÏÝÈëÁË²ÆÕþÎ£»ú¡£Ëû³Ô¾ªµØ·¢ÏÖÊÇÕÔÏ¼Ëã¼ÆÁËËû¡£Ô­À´ÕÔÏ¼ÔÚ´û¿îÉÏ×öÁËÊÖ½Å¡£
ÕÔÏ¼ÔÚÉÌÆ··ÖÅäÉÏ×öÁËÊÖ½Å¡£

33
Ñ¦ºè×î½ü×ÜÌýµ½¹ØÓÚ×Ô¼ºµÄÒ¥ÑÔ£¬ºóÀ´²Å·¢ÏÖÊÇÊ¯ÔÏÔÚµ½´¦·Ì°ùËû¡£Ëû¾ö¶¨ÕÒÊ¯ÔÏÈÏÕæµØÌ¸Ò»´Î¡£

34
Áõ¾­ÀíÈÃÑ¦»Û°ïÃ¦ÑéÊÕÒ»¸öÏîÄ¿¡£ºóÀ´ÊÇÑ¦»Û·¢ÏÖÓÐ¸ö×ÓÏîÄ¿²»´ï±ê£¬Ëýµ±¼´ÏòÁõ¾­Àí×öÁË»ã±¨¡£

35
Îâï£ºÍÁú±ÌÔÚ½ÖÉÏ±»Ò»ÈºÐ¡»ì»ìÎ§×¡¡£µ±Ê±ÊÇÁú±Ì»ú¾¯µØ²¦´òÁË110£¬´Ó¶ø±ÜÃâÁËÒ»³¡¿ÉÄÜµÄ»ìÂÒ¡£

36
ÃÀ¹¤¿ÎÉÏÎ¤ÖÎµÄÍ¬×À²»Ð¡ÐÄÉËµ½ÁË×Ô¼º¡£ÊÇÎ¤ÖÎÀä¾²Ñ¸ËÙµØ°ïËûÖ¹ÁËÑª¡£Ëû¼°Ê±µÄ´ëÊ©µÃµ½ÀÏÊ¦µÄ¿ä½±¡£
Î¤ÖÎ²»Ð¡ÐÄÉËµ½ÁË×Ô¼º¡£

37
Ð¡î£Ê®ËêÊ±£¬Ò»¸öÔ¶·¿¹ÃÄÌÄÌÈ¥ÊÀÁË¡£ËûËæ×Å¸¸Ä¸Ç°È¥µõÑäºÍ°ïÃ¦£¬ÔÚÄÇ±¯ÇººÍÃ¦ÂÒÖÐËû¸ÐÊÜµ½ÁËËÀÍö¡£

38
Ê·¾êÒâÊ¶µ½×Ô¼ºµÄ´¦¾³ÞÏÞÎ¡£ÔÚÂþ³¤µÄ°ÏÉæÖ®ºóËýÒÑ¾­½îÆ£Á¦¾¡£¬²»ÁÏÕâ×ù½¨Öþ¾¹ÊÇ²»ËÞÅ®¿ÍµÄËÂÃí¡£
Õâ×ùËÂÃí²»½Ó´ýÅ®¿Í¡£

39
¸ßÇÙµÄÓÎÏ·Õý´òµ½¹Ø¼ü´¦£¬Ö»²îÒ»²½Ëý¾Í³ÉÎª´ó¼ÀË¾ÁË¡£½á¹û¹ùç÷²»ÖªÇé°ÑµçÔ´¸ø¹ØÁË¡£

40
Íò¸Õ»ñµÃÁË±¾½ì×î¼Ñ³µÊÖµÄ³ÆºÅ¡£Ëû´øÁìµÄ³µ¶ÓÕ½¼¨²»Ë×£¬»ñµÃÁË×Ü¹Ú¾ü»¹ÆÆÁË¼ÍÂ¼¡£

41
ÑÏ×ËÍ¶Éí³´¹ÉÊÂÒµÒÑ¾­ÈýÄê¶àÁË£¬²»¹ý×î½üËýÑá¾ëÁËÕâÖÖ½ôÕÅµÄÓÎÏ·¡£ÌýËµ¹¨ÏÍ½ÓÊÖÁËËýËùÓÐµÄ¹ÉÆ±¡£

42
Ð¡Ö¾ÌìÌì³ÕÃÔÓÚÍøÂçÓÎÏ·¡£ÓÐÒ»Ìì´÷½ãÖÕÓÚÈÌÎÞ¿ÉÈÌ£¬ËýµÇÂ½ÉÏÈ¥°ÑÐ¡Ö¾´òµÄÒ»õê²»Õñ¡£

43
½ñÌìµ½×îºóÕÅÐ­ÖÕÓÚ·¢Æ¢ÆøÁË¡£²»¹ýÊÇÐèÒªÒ»ÕÅ²Í×À£¬ÇñÓñã¶ÊÇÄÜÌôÉÏÒ»ÕûÌì¡£
ËûÃÇÐèÒªµÄÊÇ²Í×À¡£

44
ÐÜ³ÉËäÈ»ÌìÐÔ¶àÒÉ£¬µ«ÊÇ¶ÔÂ½ÇåÈ´ÊÇ¾ø¶ÔµÄÐÅÈÎ¡£²»ÁÏÕâ»ØÂ½Çå¾¹È»ÆÛÆ­ÁËËû¡£

45
ÇØÖÙÊÇÒôÀÖÈ¦ÓÐÃûµÄ²®ÀÖ¡£µ±Äêºì±é´ó½ÖÐ¡ÏïµÄÎ¤æÃ£¬¾ÍÊÇÇØÖÙÊ×ÏÈ·¢ÏÖ²¢ÔÔÅàµÄ¡£

46
ÁÎÖéºÜ½ôÕÅµØ·¢ÏÖÁÖ¿µºÍãÆÀíÔÚÒ»Æð¡£×òÌìãÆÀí±ÆÆÈËýÇÔÈ¡Çé±¨£¬ËûÄÃÁÖ¿µµÄÉúÃü°²È«À´ÍþÐ²Ëý¡£

47
Ð¡À×µÄ¹¤×÷×öµÃ·Ç³£ºÃ£¬µ«ÊÇ×òÌìËû°¤ÁËÀÏ×ÜµÄÅúÆÀ¡£¾ÝËµÀÏ×Ü¶ÔËûµÄÓ¡Ïó²»´óºÃ¡£
Ð¡À×µÃµ½ÀÏ×ÜµÄ±íÑï¡£

48
¶ÔÕâ¸ö½á¹û£¬Ê·ÐÅ¸Ðµ½·Ç³£Õð¾ª¡£Ëû±¯·ßµØÖÊÎÊ×éÎ¯»áÎªÊ²Ã´£¬µ«Ã»ÓÐÈË¸æËßËû¡£


Practice Materials

Items 1
ÖÜº½°á³öÑ§Ð£Í³Ò»·ÖÅäµÄËÞÉá£¬ÔÚÍâÃæ×âÁËÒ»¸ö·¿×Ó¡£ÌýËµÊÇ²ÜÁÁÑÏÖØÓ°ÏìÁËËû¡£

2
¹ËÜ¿·¢ÏÖÁ½Ì¨¸´Ó¡»ú¶¼»µÁË£¬ºØº£µ·¹ÄÁË°ëÌìÒ²Ã»ÐÞºÃ¡£¿´À´ÏÂÎçµÄÉúÒâÊÇÎÞÂÛÈçºÎ×ö²»³ÉÁË¡£
µêÀïµÄ¸´Ó¡»ú»µµôÁË¡£

3
Öì¾¢¿ª×Å·É´¬ÔÚÒøºÓÏµÏÐ¹äÁË°ë¸öÔÂ£¬ËÀÆø³Á³ÁµÄÐÇ¼Ê¿Õ¼äÈÃËûÎÞÁÄµÃºÜ¡£Ëû¾ö¶¨ÈÃ»úÆ÷ÈË¾Í½üÕÒ¿ÅÐÇÇò×ÅÂ½¡£
Öì¾¢µÄ·É´¬ÔÚÏÉÅ®ÐÇÏµÓÎµ´¡£

4
Ëï³¿´ÓÏçÏÂ±í½ã¼Ò»ØÀ´ÓÐÒ»ÖÜÁË£¬µ«ËûÈÔÈ»ÄîÄî²»ÍüÔÚÄÇÀïµÄÃÀºÃÊ±¹â¡£ÄÇÊ±ºò±í½ãÌìÌì´øËûµ½´¦Íæ¡£

5
ÖìÑãÕýÔÚÎªÆÚÄ©¿¼ÊÔ×ö×¼±¸£¬Ëý¸Ðµ½ÓÐÒ»µãµã½ôÕÅ¡£ºóÀ´ÍõÀÏÊ¦Ç×ÇÐµØ¹ÄÀøÁËËý¡£

6
Ò»´óÇåÔç³ÂÓ¥ºÍÐ¡á¯¾Í¿ªÊ¼ÅÀ»ÆÉ½£¬ÅÀ×ÅÅÀ×ÅËû¾õµÃÓÐµã¿Ú¿Ê¡£ÕâÊ±ºòÊÇÐ¡á¯µÝ¸øËûÒ»Æ¿Ë®¡£

7
¾ÍÔÚ´ó¼Ò»¹Ã»ÓÐ¿´³ö¶ËÄßÀ´µÄÊ±ºò£¬ÊÇÁúÑïÔçÔçµØËø¶¨ÁË·½âù¡£ºóÀ´µÄÊÂÊµÖ¤Ã÷ËûÊÇÕýÈ·µÄ¡£

8
°Ö°Ö¸ø´ó¼Ò¶¼×¼±¸ÁËºÏÊÊµÄÊ¥µ®ÀñÎï£¬Î¨¶ÀÍü¼ÇÁË¼ÒÀïµÄÐ¡¹·±´±´¡£Õâ¼þÊÂÈÃÐ¡ÐÂ¸Ðµ½ºÜ²»Âú¡£

9
Ð¡Îâ¿´ÁË¼¸±¾ÎäÏÀÐ¡ËµÖ®ºó£¬·Ç³£ÏòÍù´óÏÀÃÇÓÎµ´½­ºþµÄÉú»î£¬Ëû¾ö¶¨µ½ÉÙÁÖËÂ°ÝÊ¦Ñ§ÒÕ¡£
Ð¡Îâ¾ö¶¨µ½Îäµ±É½°ÝÊ¦Ñ§ÒÕ¡£

10
ÐìÒ¯Ò¯ÕâÒ»Éú¾­Àú¿²¿À£¬µ«ÊÇËùÓÐÈÏÊ¶ËûµÄÈË¶¼ºÜ×ðÖØËû£¬ÒòÎªËûÓÐÒ»¿Å´È°®¿íÈÝµÄÐÄ¡£
